# Characterization of a Botybirnavirus Conferring Hypovirulence in the Phytopathogenic Fungus *Botryosphaeria dothidea*

**DOI:** 10.3390/v11030266

**Published:** 2019-03-17

**Authors:** Lifeng Zhai, Mengmeng Yang, Meixin Zhang, Ni Hong, Guoping Wang

**Affiliations:** 1 College of Plant Science and Technology, Huazhong Agricultural University, Wuhan 430000, China; zhailf@yeah.net (L.Z.); myang@webmail.hzau.edu.cn (M.Y.); whni@mail.hzau.edu.cn (N.H.); 2 College of Life Science and Technology, Yangtze Normal University, Chongqing 400000, China; zhangmeixin00@126.com; 3 National Key Laboratory of Agromicrobiology, Huazhong Agricultural University, Wuhan 430000, China

**Keywords:** *Botryosphaeria dothidea*, hypovirulence, *Botybirnavirus*, genome, double-stranded RNA virus

## Abstract

A double-stranded RNA (dsRNA) virus was isolated and characterized from strain EW220 of the phytopathogenic fungus *Botryosphaeria dothidea*. The full-length cDNAs of the dsRNAs were 6434 bp and 5986 bp in size, respectively. The largest dsRNA encodes a cap-pol fusion protein that contains a coat protein gene and an RNA-dependent RNA polymerase (RdRp) domain, and the second dsRNA encodes a hypothetical protein. Genome sequence analysis revealed that the sequences of the dsRNA virus shared 99% identity with Bipolaris maydis botybirnavirus 1(BmBRV1) isolated from the causal agent of corn southern leaf blight, *Bipolaris maydis*. Hence, the dsRNA virus constitutes a new strain of BmBRV1 and was named Bipolaris maydis botybirnavirus 1 strain BdEW220 (BmBRV1-BdEW220). BmBRV1-BdEW220 contains spherical virions that are 37 nm in diameter and consist of two dsRNA segments. The structural proteins of the BmBRV1-BdEW220 virus particles were 110 kDa, 90 kDa, and 80 kDa and were encoded by dsRNA1 and 2-ORFs. Phylogenetic reconstruction indicated that BmBRV1 and BmBRV1-BdEW220 are phylogenetically related to the genus *Botybirnavirus*. Importantly, BmBRV1-BdEW220 influences the growth of *B. dothidea* and confers hypovirulence to the fungal host. To our knowledge, this is the first report of a botybirnavirus in *B. dothidea*.

## 1. Introduction

Mycoviruses have been reported from a wide range of fungal species [1,2,3,4]. The majority of mycoviruses reported to date consist of double-stranded RNA (dsRNA) and positive-sense single-stranded RNA (+ssRNA), whereas the rest possess negative-sense single-stranded RNA (-ssRNA) and single-stranded DNA (ssDNA) as their genetic material [1,4,5,6,7,8,9,10]. DsRNA mycoviruses are classified into seven families, namely, *Totiviridae*, *Chrysoviridae*, *Megabirnaviridae*, *Partitiviridae*, *Quadriviridae*, *Reoviridae*, and *Endornaviridae* [1,4]. A proposed dsRNA virus family *Botybirnaviridae* has been described, which includes the newly reported bipartite dsRNA virus Botrytis porri botybirnavirus 1 (BpRV1) [11]. Currently, the family *Botybirnaviridae* includes six members, namely, BpRV1, Sclerotinia sclerotiorum botybirnavirus 1 (SsBRV1), SsBRV2, Alternaria botybirnavirus 1 (ABRV1), Bipolaris maydis botybirnavirus 1 (BmBRV1), and Soybean leaf-associated botybirnavirus 1 (SlaBRV1) [11,12,13,14,15,16]. Viruses of the family *Botybirnaviridae* consist of two dsRNAs. A satellite-RNA associated SsBRV1 has been identified [12], whereas no satellite RNA was detected in other botybirnavirus-infected strains. In general, mycoviruses cause mild or no obvious effects on their hosts [1], but some can increase mycelial growth [17], reduce virulence, and suppress the growth of their hosts [3,4,11,18,19,20,21,22,23,24,25]. The hypovirulence-associated mycoviruses have an exploitable potential for controlling diseases and thus may be utilized as biocontrol agents [3,4,18].

*Botryosphaeria dothidea* is capable of infecting a broad range of hosts [26,27]. The pathogen causes stem warts, stem cankers, and fruit rot diseases in apple, pear, and grape trees in China [28,29,30,31]. Mycoviruses infecting *B. dothidea* have recently been isolated and characterized [21,24,32,33]. Botryosphaeria dothidea chrysovirus 1 (BdCV1) and Botryosphaeria dothidea RNA virus 1 (BdRV1) are associated with hypovirulence in the fungal host [21,24,32]. Botryosphaeria dothidea victorivirus 1 (BdV1) and Botryosphaeria dothidea partitivirus 1 (BdPV1) have no obvious effects on the growth of the host [21,33].

Here, we report a dsRNA virus isolated from *B. dothidea* strain EW220. The dsRNA virus exhibited a similar genomic structure to BmBRV1 and possessed 99% nucleotide sequence identity with BmBRV1. Therefore, the dsRNA virus was designated as Bipolaris maydis botybirnavirus 1 strain BdEW220 (BmBRV1-BdEW220). BmBRV1-BdEW220 confers hypovirulence to the host *B. dothidea* and can be transmitted via asexual sporulation and hyphal anastomosis. Based on these findings, BmBRV1-BdEW220 might have a potential use as a biological control agent for diseases caused by *B. dothidea*.

## 2. Materials and Methods

### 2.1. Fungal Isolates and Culture Conditions

Six strains of *B. dothidea* were used in this study (Table 1). Strain EW220 was isolated from a wart-diseased stem tissue of pear (*Pyrus bretschneideri* cv. Suli) grown in Hubei Province in China. Strain JNT1111, which is highly virulent to pear fruits and shoots [30], was isolated from Shanxi Province in China and used as a control in assessing the biological features in this study. The virus-free strain EW220-64 constitutes a single-conidium isolate of strain EW220. Strains EW220-64-T1, EW220-64-T2, and EW220-64-T3 from EW220-64 were obtained from a horizontal transmission assay. The strains were cultured on potato dextrose agar (PDA) plates at 28 °C in the dark. Mycelial agar discs (5 mm) were stored in sterile 25% glycerol solution at −80 °C. Strains EW220 and JNT1111 were identified by ITS sequence analysis using the ITS1 and ITS4 primers. The ITS sequences of strains EW220 and JNT1111 were deposited in GenBank under accession numbers KC960983 and MK581056, respectively.

### 2.2. Extraction and Purification of dsRNA

The strains were cultured on cellophane membranes overlaid on the surfaces of PDA plates for 5 days at 28 °C in the dark. Approximately 0.5 g of fresh mycelium from each strain was harvested and ground into a fine powder in liquid nitrogen. DsRNAs were extracted using a patented method (no. ZL201310072994.3). Briefly, the method was developed by using silica columns combined with the application of a tissue lysis solution containing sodium dodecyl sulphate (SDS, 2%), as well as binding and washing with buffers containing highly concentrated guanidinium thiocyanate (GuSCN, 50% *w*/*v*). The resulting dsRNA extract was dissolved in a total of 50 µL RNase-free water. The dsRNA samples were digested with DNase I (RNase-free) and S1 nuclease (TaKaRa, Dalian, China) to eliminate any contaminating DNA and ssRNA. The purified dsRNA samples were subjected to electrophoresis in a 1% (*w*/*v*) agarose gel and viewed on a UV transilluminator after staining with 0.1 μg/mL ethidium bromide. The separated dsRNA segments were excised from the gel and purified using a DNA gel extraction kit (Axygen Scientific, Inc., Wujiang, China). The purified dsRNAs were dissolved in diethylpyrocarbonate (DEPC)-treated water and kept at −80 °C.

### 2.3. cDNA Synthesis and Molecular Cloning

The purified dsRNAs (1.0 µg) combined with 0.1 μg random hexamer primers mixture d(N)6 (TaKaRa, Dalian, China) were used in the first-strand cDNA synthesis using the Moloney murine leukemia virus (M-MLV) reverse-transcriptase according to the manufacturer’s instructions (Fermentas, Madison, WI, USA). The second-strand cDNAs were synthesized as previously described in Reference [34], purified by chloroform and isoamyl alcohol, and A-tailed by co-incubation with Taq DNA polymerase and dNTPs (TaKaRa, Dalian, China). The resulting double-stranded cDNAs were ligated into the pMD18-T vector (TaKaRa, Dalian, China) and transformed into competent cells of *Escherichia coli* DH5 α. All of the positive clones with inserts of more than 500 bp in size were sequenced. Sequence gaps between clones were determined by reverse transcription-polymerase chain reaction (RT-PCR) using primers designed from the obtained cDNA sequences. The terminal sequence of each of the dsRNAs was obtained according to a ligase-mediated rapid amplification of cDNA ends (RLM-RACE) procedure described in [35]. Sequencing was performed by Genscript Biotechnology Co., Ltd., Nanjing, China. At least three independent clones of each product were determined in both orientations.

### 2.4. Sequence Analysis, Alignment, and Phylogenetic Analysis

The DNAMAN software package (DNAMAN version 6.0; Lynnon Biosoft, Montreal, Canada) was used to detect potential open reading frames (ORFs). The prediction of the stem-loop structures of the terminal sequences of the viral RNAs was conducted using the RNA folding program from the Mfold website, implementing the default parameters (http://mfold.rna.albany.edu/?q=mfold/RNA-Folding-Form2.3) [36]. The CD-search website of the National Center for Biotechnology Information (NCBI) (http://www.ncbi.nlm.nih.gov/) and the motif scan website (http://www.genome.jp/tools/motif/) were used to identify the conserved domains of the full-length cDNA virus sequences. MAFFT software [37] was used for multiple nucleotide and amino acid sequence alignments, and the results were visualized on the BoxShade website (http://www.ch.embnet.org/software/BOX_form.html). Phylogenetic trees were constructed using the maximum likelihood method in Molecular Evolutionary Genetics Analysis (MEGA) software 7 [38]. Bootstrap values (relative) were generated based on 1000 replicates. Reference sequences of the viruses used for comparative analyses were obtained from NCBI (http://www.ncbi.nlm.nih.gov/genomes).

### 2.5. Virion Purification

The purification of viral particles was performed as previously described [24]. Strain EW220 was grown at 28 °C for 10 days on sterile cellophane films placed on PDA. About 20 g mycelia was harvested and ground into a fine powder in liquid nitrogen. The powder was transferred to a container with 120 mL of 0.1 M sodium phosphate extraction solution (pH 7.0). The suspension liquid was centrifuged at 10,000× *g* for 20 min. The supernatant was transferred to another plastic tube and then centrifuged at 10,000× *g* for 20 min again to remove any remaining hyphal cell debris. The supernatant was centrifuged at 100,000× *g* at 4 °C for 2 h. The obtained pellet was re-suspended in 0.4 mL of 0.1 M phosphate buffer (PB, pH 7.0). The supernatant containing the virus particles was then overlaid on a centrifuge tube containing sucrose gradient (10–50%, *w*/*v*) and centrifuged at 70,000× *g* at 4 °C for 3 h. Each fraction was individually collected, and total RNA was extracted using phenol-chloroform and chloroform-isoamyl alcohol. Viral dsRNAs were detected by 1% agarose gel electrophoresis. The fractions containing viral dsRNAs were centrifuged at 100,000× *g* at 4 °C for 2 h, and the precipitate was suspended in 100 μL PB (0.05 M, pH 7.0). Viral particles were stained with 2% (*w*/*v*) uranyl acetate and examined by transmission electron microscopy (Model Tecnai G2 20; Field Electron and Ion Company, Hillsboro, OR, USA). The viral particle suspension was loaded onto a 12% polyacrylamide gel amended with 1% (*w*/*v*) sodium dodecyl sulfate (SDS), run for 8 h at 20 V/cm, and then stained with Coomassie brilliant blue R-250 (Bio-Rad, Hercules, CA, USA). The separated proteins in the SDS-PAGE were used for polypeptide mass fingerprinting-mass spectrum (PMF-MS) analyses.

### 2.6. Vertical and Horizontal Transmission

Single-conidium sub-strains from the strain EW220 were used for evaluating the vertical transmission of the mycovirus. Conidia from strain EW220 were induced on a PDA plate as previously described [30]. The individual sub-strains were obtained as described previously [24], and 120 individual single-conidium sub-strains were assessed for the presence of dsRNA segments in the mycelia. Horizontal transmission of viral dsRNA segments via hyphal anastomosis was executed according to the previously described method [39]. The strain EW220 served as the donor, and strains JNT1111 and EW220-64, which lacked detectable dsRNAs (Table 1), served as the recipients. The strain EW220 and each virus-free strain were co-cultured on the same PDA plates in the three replicates. After 5 days of co-incubation at 25 °C, the mycelial agar discs most distant from the contact point between the two colonies were obtained from the edge of the recipient strains. The new derivatives from strains JNT1111 (EW220-J11-T1, EW220-J11-T2, and EW220-J11-T3) and EW220 (EW220-64-T1, EW220-64-T2, and EW220-64-T3) were individually purified by single hyphal tip culturing and used to assess the mycovirus content and biological properties.

### 2.7. Biological Testing

Using previously described procedures [30], the mycelial growth rate, colony morphology, and virulence of the virus-infected (EW220, EW220-64-T1, EW220-64-T2, and EW220-64-T3) and virus-free (EW220-64 and JNT1111) strains of *B. dothidea* were assessed (Table 1). Briefly, mycelial growth rates of the strains were measured on fresh PDA plates at 28 °C. Each of the strains was assessed using six replicates. To evaluate the virulence of strain EW220, 12 one-year-old shoots on five-year-old pear (*P. pyrifolia* cv. ‘Hohsui’) plants grown in the field were used. The strains EW220 and JNT1111 were tested for their pathogenicity on unwounded shoots by directly placing colonized agar plugs (5 mm in diameter) on the surface of the lenticels (shoots disinfected with 75% ethanol). Wart and canker symptoms were monitored, and disease incidence was recorded at 60 days post inoculation (dpi). To assess the virulence of all the tested strains, actively growing mycelial plugs from each strain were inoculated onto four detached pear fruits (*P. bretschneideri* cv. ‘Huangguan’). A PDA plug lacking the fungi was used as the non-inoculated control. Inoculated fruits were maintained in a 28 °C incubator for 4 days, following which the average lesion size was measured. The experiment was repeated twice. The biological properties were investigated by one-way analysis of variance (ANOVA) using the SAS 9.0 program, and differences with *p*-values < 0.05 were considered statistically significant.

## 3. Results

### 3.1. Biological Characteristics of Strain EW220 and the Detection of dsRNA

**Figure 1:**
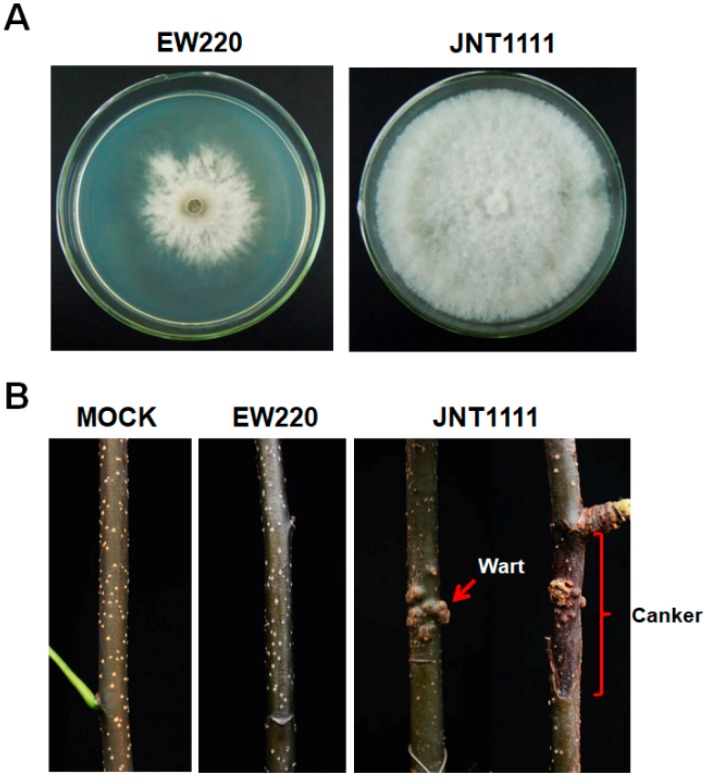
Colony morphology and virulence of strains EW220 and JNT1111 on pear shoots (*P. pyrifolia* cv. ‘Hohsui’). (**A**) Colony morphology in potato dextrose agar (PDA) medium (28 °C, 4 days); (**B**) pear shoots unwound-inoculated with colonized plugs of the tested strains photographed at 60 dpi.

Strain EW220, which was cultured on PDA plates at 28 °C in the dark, exhibited abnormal growth. The mycelia grew slowly and exhibited a few aerial hyphae (Figure 1A). The developing colonies covered the entire dish (90-mm diameter) at 10 dpi. In contrast, the virus-free strain JNT1111 grew rapidly and developed numerous hyphae. The colony of strain JNT1111 could cover the entire culture dish at 3 dpi. The growth rate of the strain EW220 was only 30% of that of the strain JNT1111 (Figure 1A). The annual branches of the pear tree (*P. pyrifolia* cv. ‘Hohsui’) inoculated with strain EW220 showed no symptoms at 60 dpi (Figure 1B). However, the strain JNT1111 induced typical warts and canker symptoms (Figure 1B), and the disease incidence reached 80% (10 out of 12 inoculate sites). These results suggested that strain EW220 was a weak virulent strain.

**Figure 2:**
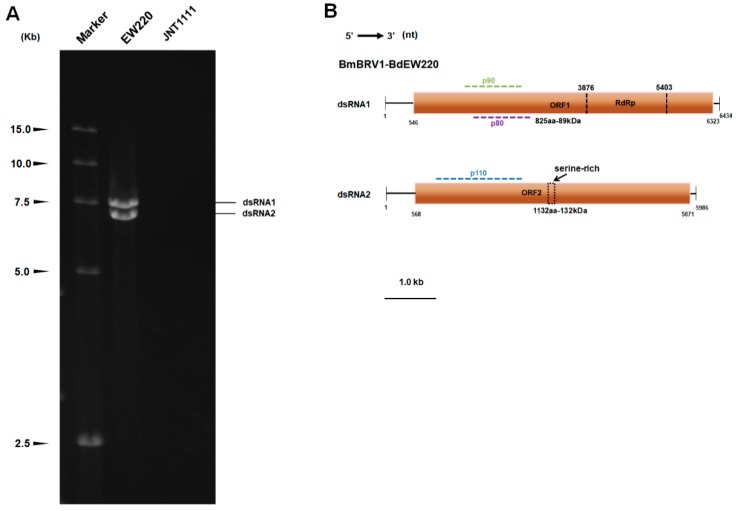
Double-stranded RNAs extracted from strain EW220 and the genomic organization of Botryosphaeria dothidea botybirnavirus 1 (BmBRV1-BdEW220). (**A**) One percent agarose gel electrophoretic profiles of dsRNA preparations extracted from strains EW220 and JNT1111 after digestion with DNase I and S1 nuclease; (**B**) schematic diagrams of the genomic organization of BmBRV1-BdEW220.

DsRNA was extracted from strains EW220 and JNT1111. The results of the 1.0% agarose gel electrophoresis showed that strain EW220 harbored dsRNA segments with sizes ranging from 7.0 kb to 7.5 kb. No dsRNAs were detected in the preparation from strain JNT1111 using the same treatment conditions (Figure 2A).

### 3.2. Genetic Analysis of dsRNAs

Based on the ds-cDNAs library, the 7.5-kb dsRNA generated 23 random fragments with lengths ranging from 800–2200 bp. BLASTn analysis of these fragments was highly similar to segment 1 of BmBRV1 (sequence identities ranging from 99% to 100%). The 7-kb dsRNA generated a total of 18 random fragments with lengths ranging from 800 bp to 2000 bp. BLASTn analysis revealed that these sequences were most similar to segment 2 of BmBRV1 (sequence identities ranging from 99% to 100%). By combining the sequences obtained from the RT-PCR and RLM-RACE, we obtained the cDNA sequences of the two segments of the dsRNAs. The full-length cDNAs of dsRNAs were 6434 bp, and 5986 bp, respectively (Figure 2B). The corresponding sequences were deposited in GenBank under accession numbers MH684534 and MH684535.

The cDNA length of dsRNA1 was 6434 bp with a GC content of 49.4%. BLASTn searches revealed that the dsRNA1 sequence shared high similarity (E-value = 0.0; 99% identity; 6381/6434 nt) with the dsRNA1 of BmBRV1 (Figure S1). A single large ORF from positions 546 nt to 6323 nt was discovered, and this ORF encodes a tentative protein (P1) of 1925 amino acid (aa) residues with a mass of approximately 217 kDa. Motif scanning revealed that the P1 contained a conservative RdRp functional domain (RdRp_4 Pfam02123) at its C-terminal (Figure 2B). BLASTp analysis indicated that the protein was closely related to the cap-pol fusion protein of the members of genus *Botybirnavirus*, exhibiting 30% to 99% identity. In particular, P1 showed significant similarity to the cap-pol fusion protein of BmBRV1, which showed 99% identity (1906/1925 aa). The protein P1 was also most closely related to the cap-pol fusion protein of SsBRV1 with an identity of 82% (Figure S2). However, two amino acid fragments from 1 to 240 and from 901 to 1080 of BmBRV1-BdEW220 exhibited low similarity with SsBRV1 (56% and 50% identities, respectively) (Figure S2). Interestingly, an amino acid sequence (107 aa) encoded by the nucleotide sequence of dsRNA1 (from 3462 to 3785 nt; Figure S1) showed only 39% identity with SsBRV1 (Figure S2). In addition, two bipartite nuclear localization signal profiles from 1128 to 1142 aa (KRAVYTIGTLLRKLK, *E* value = 2.1 × 10^−4^) and from 1417 to 1431 aa (KRNSQLLEEKEERRR, *E* value = 2.1 × 10^−4^), a phosphatase tensin-type domain profile from 1264 to 1453 aa (*E* value = 1.7 × 10^−2^), a glutamine cyclotransferase from 1333 to1346 aa (KLKSLGLKVDGHAN, *E* value = 0.0026), and an immunoreceptor tyrosine-based activation motif from 420 to 440 aa (TDLYNSIGDRAIAERYYDHVVTA, *E* value = 0.69) were also detected in the P1.

The total length of the cDNA of dsRNA2 was 5986 bp with a GC content of 50.6%. BLASTn analysis shows that the sequence was most similar to BmBRV1 segment 2 (Figure S2), showing 98% identity (*E* value = 0.0; 5886/5986 nt). DsRNA2 also harbored a single ORF (ORF2, nt 568–5671) on the genomic plus strand RNA. The protein (P2), which consisted of 1767 amino acid residues and was encoded by dsRNA2-ORF, was determined to have a molecular mass of 197 kDa (Figure 2B). A BLASTp search of P2 showed significant similarity to the hypothetical proteins of the members of genus *Botybirnavirus*, including BmBRV1 (99% identity; 1741/1767 aa), SsBRV1 (74% identity), ABRV1 (42% identity), SlaBRV1 (40% identity), SsBRV2 (28% identity), and BpRV1 (28% identity). Similarly, a nucleotide fragment of BmBRV1-BdEW220 from 3630 to 4326 nt had low similarity with SsBRV1 (58% identity, Figure S3). The protein sequences (232 aa) encoded by this region were similar to a region of the hypothetical proteins, from 1022 to 1243 aa of SsBRV1, and exhibited 41% identity (Figure S4). In addition, an adenovirus GP19K from 18 to 41 aa (LLGCTSMLFVEKTRGGVNLKKKMP, *E* value = 0.0025) and a serine-rich region profile from 1039 to 1084 aa (SNQSDADSDSDSGLARKSKPQQSLAKNLSSLKDSESESASSSDDES, *E* value = 1.3) were also found in P2 (Figure 2B).

**Figure 3:**
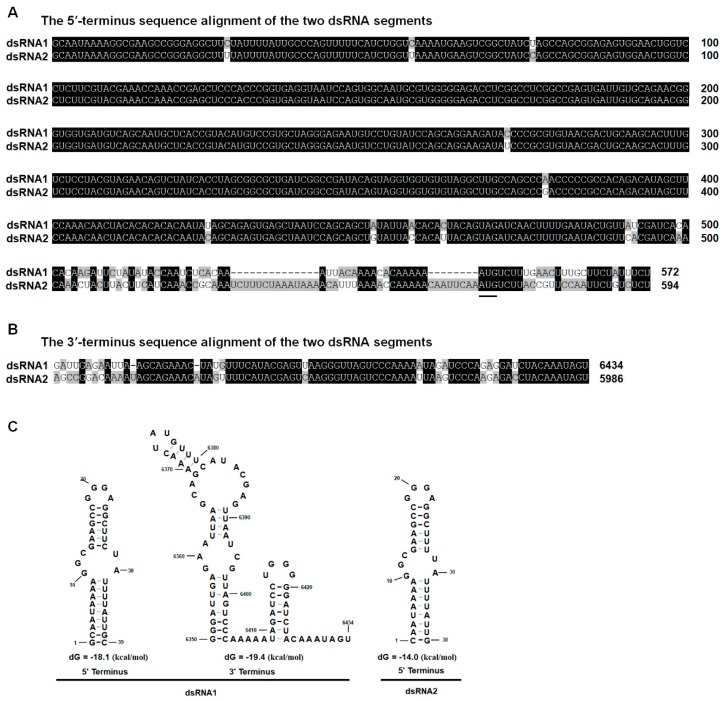
Multiple sequence alignments and predicted secondary structures for the terminal regions of the coding strand of dsRNA1 and dsRNA2. (**A**) Conserved sequences of the 5′-UTRs of dsRNA1 and dsRNA2; (**B**) Conserved sequences of the 3′-UTRs of dsRNA1 and dsRNA2; the conserved sequences are shown in black; (**C**) Predicted secondary structures for the 5′- and 3′-terminus of the coding strands of dsRNA-1 and dsRNA-2 of BmBRV1-BdEW220.

The 5′-untranslated regions (UTR) of the coding strands of dsRNA1 and dsRNA2 were 545 bp and 567 bp in length, respectively. Similarly, the 5′-UTRs of BmBRV1, BpRV1, ABRV1, SsBRV1, and SsBRV2 were 546/568 bp, 404 bp/405 bp, 544 bp/594 bp, 568 bp/577 bp, and 412 bp/411 bp in length, respectively [11,12,13,14,15]. The nucleotide sequences (about 498 bp) close to the 5′-terminus of dsRNA 1 and dsRNA 2 shared 97.8% sequence identity with each other (Figure 3A). The corresponding 3′-UTRs were 111 bp and 115 bp in length, respectively, and the nucleotide sequences (about 71 bp) close to the 3′-terminus of dsRNA 1 and dsRNA 2 shared 84.3% sequence identity (Figure 3B). The secondary structures of the 5′- and 3′-UTRs of dsRNA 1 and dsRNA 2 were predicted to fold a stable stem-loop structure (Figure 3C). However, the stem-loop structure at the 3′-UTRs of dsRNA 2 was not detected.

According to the International Committee on Taxonomy of Viruses (ICTV), a sequence identity >90% is indicative that the compared sequences belong to the same virus species [40]. Therefore, we can conclude that the viral sequence analyzed in this study is a strain of BmBRV1. It was described as Bipolaris maydis botybirnavirus 1 strain BdEW220 (BmBRV1-BdEW220).

### 3.3. Phylogenetic Analysis of the dsRNA Virus

**Figure 4:**
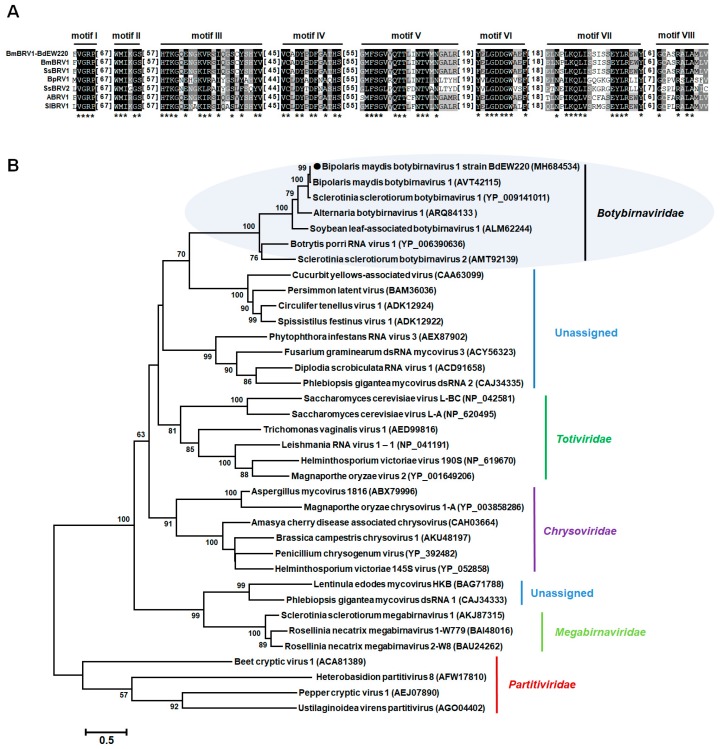
Multiple alignments of the amino acid sequences of the RNA-dependent RNA polymerase of BmBRV1-EW220 with other selected members in *Botybirnavirus* (**A**) and phylogenetic analysis of BmBRV1-EW220 (**B**). The phylogenetic trees were constructed based on the best-fit model of protein evolution (LG + G + I + F). The gamma value was 2. Bootstrap values (relative) generated using 1000 replicates are shown on the branches. Only bootstrap values of ≥50% are presented, and the branch lengths are proportional to the genetic distances. The black circle represents BmBRV1-EW220. Abbreviations used in Figure 4A: BmBRV1-EW220, Bipolaris maydis botybirnavirus 1 strain BdEW220; BmBRV1, Bipolaris maydis botybirnavirus 1; SsBRV1, Sclerotinia sclerotiorum botybirnavirus 1; SsBRV2, Sclerotinia sclerotiorum botybirnavirus 2; ABRV1, Alternaria botybirnavirus 1; SlBRV1, Soybean leaf-associated botybirnavirus 1; BpRV1, Botrytis porri RNA virus 1.

Multiple amino acid alignments of the predicted RdRp indicated the existence of the motifs I–VIII in BmBRV1-BdEW220 and other members of the family *Botybirnaviridae* (Figure 4A). The phylogenetic tree was constructed based on the amino acid sequences of RdRp encoded by members of the families *Totiviridae*, *Partitiviridae*, *Chrysoviridae*, *Quadriviridae*, and *Megabirnaviridae*, as well as some unassigned dsRNA viruses. The results indicated that the members of the family *Botybirnaviridae* grouped into two distinct clusters. BmBRV1-BdEW220 clustered together with BmBRV1, SsBRV1, ABRV1, and SlBRV1 to form a separate evolutionary clade from the other cluster, which contained BpRV1 (*Botybirnaviridae* mode) and SsBRV2 (Figure 4B).

### 3.4. Virus Particles

**Figure 5:**
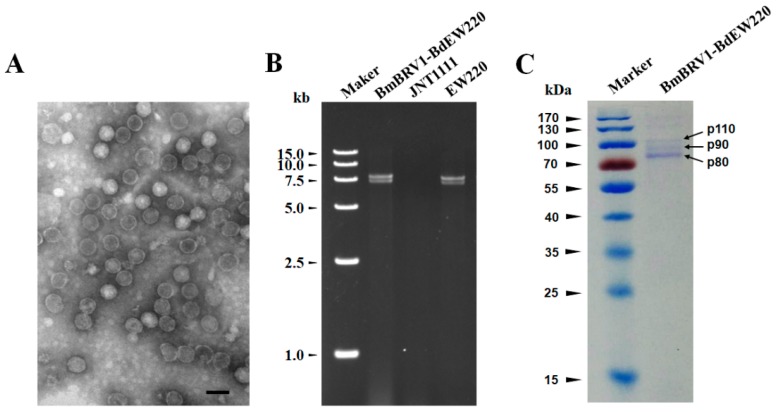
Features of the BmBRV1-BdEW220 viral particles (**A**) Transmission electron microscopy (TEM) images of the BmBRV1-BdEW220 viral particles; (**B**) agarose gel electrophoresis of the dsRNAs extracted from purified virus particles of BmBRV1-BdEW220 and the mycelia of strain EW220 (line EW220); (**C**) SDS-PAGE analysis of the purified viral particles shows the three distinct protein bands. The scale bar represents 50 nm.

Virus particles were purified from the mycelia of strain EW220 using sucrose gradient (10–50% sucrose gradient fractions) centrifugation. Agarose gel electrophoresis of the nucleic acids extracted from the sucrose fractions showed that the dsRNA segments were mostly recovered from the 40% fraction (data not shown). To examine the morphology of the viral particles of BmBRV1-BdEW220, the fraction containing viral dsRNAs was centrifuged and re-suspended. The viral particles were isometric and approximately 37 nm in diameter as observed under TEM (Figure 5A). Furthermore, the result of the agarose gel electrophoresis showed that the dsRNAs directly extracted from the purified viral particles and extracted from the mycelia of strain EW220 had similar migration rates (Figure 5B). In addition, SDS-PAGE electrophoresis of the viral particles revealed three major protein bands with approximate sizes of 110 kDa (p110), 90 kDa (p90), and 80 kDa (p80) (Figure 5C).

### 3.5. Structural Proteins of the Virus

To further assess the virus structural proteins, the proteins of the BmBRV1-BdEW220 viral particle preparations were loaded onto SDS-PAGE gels and separated (Figure 5C). These were individually assessed, and their corresponding genes were determined using PMF-MS. A total of 13, 8, and 11 peptide fragments were discovered in p110, p90, and p80, respectively (Tables S1–S3). The 110 kDa protein matched the partial sequence at amino acid 132–685 of P2 encoded by dsRNA2, accounting for 31.0% of the entire coverage (1767 amino acids). The 90 kDa protein matched the partial sequence at amino acid 323–865 of P1 encoded by dsRNA1, accounting for 28.0% of the entire coverage (1925 amino acids). The 11 peptides from p80 corresponded to an ORF1-encoded protein at the amino acid position of 383–865, accounting for 25.0% of the complete coverage (1925 amino acids). Based on the PMF-MS results, p110 was confirmed to correspond to the deduced 110-kDa proteins encoded by the ORF2 of dsRNA2, and p90 and p80 were encoded by the ORF1 of dsRNA1 (Figure 2B, Tables S1–S3). Therefore, the structural proteins of BmBRV1-BdEW220 are derived from two ORFs encoded by dsRNA1 and dsRNA2.

### 3.6. Vertical and Horizontal Transmission of BmBRV1-BdEW220

**Figure 6:**
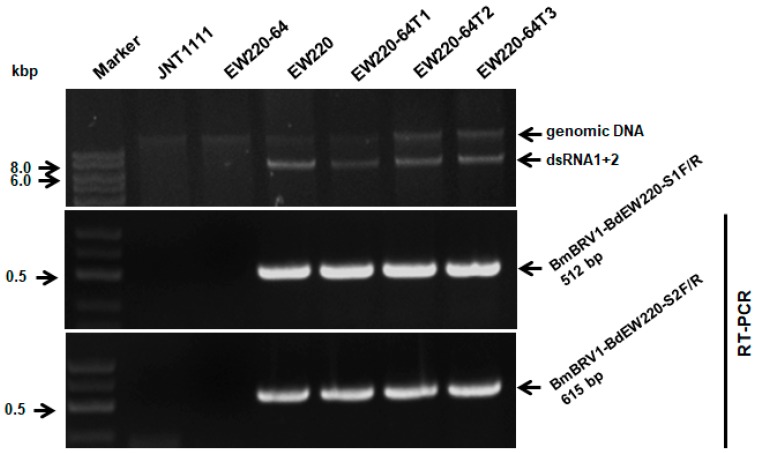
Detection of BmBRV1-BdEW220 in different strains of *B. dothidea* by dsRNAs profiling and RT-PCR with specific primers. Strains EW220-64-T1, EW220-64-T2, and EW220-64-T3 were derived from EW220-64 in the pairing cultures of EW220/EW220-64. The primers BmBRV1-BdEW220-S1F (5′-GCGCTGAGTGGATGATCAAAG-3′) and BmBRV1-BdEW220-S1R (5′-CTCTTCGTCTGGCAAAAAGCC-3′) were used for detecting the BmBRV1-BdEW220-dsRNA1 segment, and BmBRV1-BdEW220-S2F (5′-GCACTAAGAGAGACTTTCGAG-3′) and BmBRV1-BdEW220-S2R (5′-CGGTAGGATC ATCCATAGTG-3′) were used for detecting the BmBRV1-BdEW220-dsRNA2 segment.

One hundred and twenty single-conidium strains were derived from strain EW220. Detection of dsRNAs revealed that strain EW220-64 was not infected by BmBRV1-BdEW220 (Figure 6), whereas others harbored dsRNA segments. This result revealed that BmBRV1-BdEW220 in strain EW220 was vertically transmitted to the conidia sub-strains. Furthermore, the dsRNA-free strains JNT1111 and EW220-64 were used as the recipients in the horizontal transmission. For each strain, three derivative sub-strains were obtained. The dsRNAs of BmBRV1-BdEW220 were extracted from the derivatives of the recipient strains. These results suggest that BmBRV1-BdEW220 from strain EW220 was horizontally transmitted to strains EW220-64T1, EW220-64T2, and EW220-64T3 via hyphal contact (Figure 6). However, BmBRV1-BdEW220 from strain EW220 was not horizontally transmitted to the sub-strains from strain JNT1111 (data not shown). These results revealed that dsRNAs of BmBRV1-BdEW220 can be successfully transmitted into the single-conidium strain EW220-64 but not into strains with different of origins, such as JNT1111, as earlier proposed for BpRV1 and SsBRV1 [11,12].

### 3.7. Influence of BdBRV1 on the Biological Properties of B. dothidea

**Figure 7:**
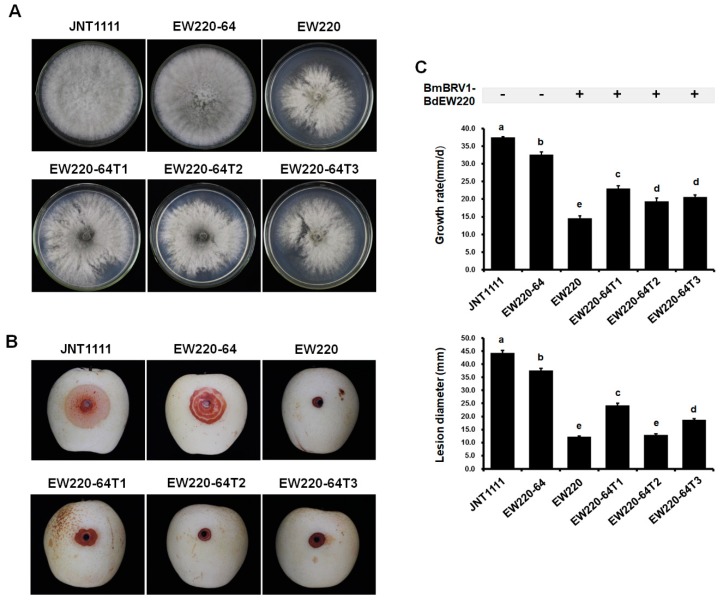
Colony morphology and virulence of strain EW220, strain JNT1111, and derived sub-strains on pear fruit (*P. bretschneideri* cv. Huangguan). Strains EW220-64-T1, EW220-64-T2, and EW220-64-T3 were derived from EW220-64 in the pairing cultures of EW220/EW220-64. (**A**) Colony morphology in PDA medium (28 °C, 3 days); (**B**) pear fruits wound-inoculated with colonized plugs of tested strains and photographed at 4 dpi; (**C**) statistical analysis of the growth rate and the lesion size. The error bars indicate the standard deviations from different sample means. The letter indicates a significant difference at the *p* < 0.05 level of confidence based on a multiple range test.

Biological assessment of the two BmBRV1-BdEW220-free and four BmBRV1-BdEW220-infected strains (Figure 6), including colony morphology, growth rate, and virulence, was performed. On PDA plates, the colonies of strains JNT1111 and EW220-64 exhibited radial growth and thick hyphae (Figure 7A). Compared to strains JNT1111 and EW220-64, the derivative strains (EW220-64T1, EW220-64T2, and EW220-64T3) grew slower on PDA at 28 °C and formed abnormally whitish compact colonies (Figure 7A). The average growth rate of each tested strain varied from 14.6 mm/day to 37.5 mm/day. The average growth rate of the BmBRV1-BdEW220-infected strains EW220 (14.6 mm/day), EW220-64T1 (23.0 mm/day), EW220-64T2 (19.4 mm/day), and EW220-64T3 (20.7 mm/day) were significantly lower than that of EW220-64 (32.6 mm/day), which was the single-conidium strain from EW220 (Figure 7C). These results revealed that BmBRV1-BdEW220 exerted greater influence on the growth rate of *B. dothidea*. The results of the pathogenicity tests on the pear fruit revealed that all of the tested strains caused rot lesions on the pear fruits. The average diameter of the lesions induced by strain EW220-64 (37.6 mm) was significantly larger than those induced by the strains EW220 (12.3 mm), EW220-64T1 (24.3 mm), EW220-64T2 (12.9 mm), and EW220-64T3 (18.8 mm) (Figure 7C). Furthermore, the largest lesion was observed in JNT1111 (44.4 mm) (Figure 7B,C). In combination, these findings indicate that BmBRV1-BdEW220 confers hypovirulence to the fungal host *B. dothidea*.

## 4. Discussion

This study presents the first report of a molecular and morphological characterization of a botybirnavirus isolated from the plant phytopathogenic fungus *B. dothidea*. Based on the sequence analysis of the virus, it constitutes a strain of BmBRV1 and infects *B. maydis*. It was named Bipolaris maydis botybirnavirus 1 strain BdEW220 (BmBRV1-BdEW220). The largest dsRNA of BmBRV1-BdEW220 encodes a cap-pol fusion protein that contains a coat protein gene with an RNA-dependent RNA polymerase (RdRp) domain, and the second dsRNA encodes a hypothetical protein. BmBRV1-BdEW220 contains spherical virions that are 37 nm in diameter. The viral particle of BmBRV1-BdEW220 is composed of different structural proteins. BmBRV1-BdEW220 influences the growth of *B. dothidea* and confers hypovirulence to the fungal host.

Our research is the first to show that botybirnavirus occurs naturally in two taxonomically distinct fungi. The host of BmBRV1 is *B. maydis* [16], which is the causal agent of corn southern leaf blight [41]. In fact, *B. dothidea*, the host of BmBRV1-BdEW220, usually infects woody plants [24,25]. They have different hosts and biotopes. Even though *B. maydis* and *B. dothidea* belong to Dothideomycetes, it is obvious that these two fungi are not phylogenetically closely related. For instance, *B. dothidea* belongs to *Botryosphaeriales*, whereas *B. maydis* belongs to Pleosporales. In another report, a conspecific mycovirus that naturally occurs in two taxonomically distinct fungi, namely, Ophiostoma novo-ulmi mitovirus 3a-Ld (OnuMV3a-Ld), was found in *Sclerotinia homoeocarpa* and *Ophiostoma novo*-*ulmi* [42]. The natural occurrence of BmBRV1 in these two fungi suggests that the horizontal transmission of this virus may have occurred between *B. dothidea* and *B. maydis*. However, the horizontal transmission experiments in this study indicated that BmBRV1-BdEW220 was successfully transmitted from strain EW220 into EW220-64, which is the single-conidium strain from EW220, but it did not transmit into a strain with different origins (strain JNT1111), as proposed before for BpRV1 [11]. These results indicated that the virus might not overcome the vegetative incompatibility in different strains. There might be another important mechanism that allows for the successful transmission of BmBRV1-BdEW220 between *B. dothidea* and *B. maydis*. In contrast to plant and animal pathogenic viruses, natural vectors are largely unknown in fungal viruses, which typically have no extracellular phase [2]. However, one of the best-known examples, SsHADV-1, infects a mycophagous insect and utilizes it as a transmission vector [43]. Further studies are needed to elucidate if there is potential insect vector transmission of BmBRV1-BdEW220 in nature.

The natural host ranges of mycoviruses have been considered to be limited to individuals within the same or closely related vegetative compatibility groups [18]. Generally, viral transmission is described in the same species by hyphal anastomosis horizontally and via spores vertically [44]. The protoplasts of several fungi have been successfully transfected with purified virus particles mediated by polyethylene glycol (PEG); the assay allowed for the extension of the host range of some mycoviruses. For instance, Sclerotinia sclerotiorum hypovirulence-associated DNA virus 1 (SsHADV-1) can infect *Sclerotinia minor* and *S. nivalis*, which are the sister species of *S. sclerotiorum* [23]. Another study showed that Aspergillus thermomutatus chrysovirus 1 (AthCV1), isolated from *Aspergillus thermomutatus,* can infect *A. fumigatus*, *A. nidulans*, and *A. niger* [45]. In vitro experiments have shown that mycoviruses are transmitted horizontally between fungal species via hyphal anastomosis [46,47]. Melzer et al. [46] described that a dsRNA virus was transmitted from isolates of *S. sclerotiorum* to *S. minor*. Cryphonectria hypovirus 1 (CHV-1) could transmit from *Cryphonectria parasitica* to an unidentified Cryphonectria species in vitro [47]. In nature, interspecies fungi in some genera could be infected by the same mycovirus. CHV-1 was detected in the strains of *C. parasitica* and *Cryphonectria* species [47], while Heterobasidion RNA virus 1 (HetRV1) occurred in five different *Heterobasidion* species [48]. In these studies, conspecific viruses have occurred during transmission in different fungal host species belonging to the same genus. Interestingly, OnuMV3a-Ld and BmBRV1-BdEW220 were found in different taxonomically-distinct fungi. Considering the high probability of co-infection of plants with phytopathogenic fungi in nature, we believe that the cross-infections of conspecific mycoviruses to fungi are not merely isolated cases and could occur more frequently.

Based on the results of the phylogenetic reconstruction, the known viruses in the family *Botybirnaviridae* comprise two different groups (Figure 4B). BmBRV1-BdEW220 and BmBRV1 were more closely related to SsBRV1 than BpRV1 [15]. The nucleotide sequences of dsRNA1 and dsRNA2 of BmBRV1-BdEW220 were similar to the corresponding dsRNAs of SsBRV1, with 77% and 76% sequence identities, respectively [15]. In contrast, some regions of the nucleotide sequences of BmBRV1-BdEW220 indicated lower identity (approximately 40%) with SsBRV1 (Figures S1 and S3). The 5′-terminus of dsRNAs (about 498 bp) of BmBRV1-BdEW220 and BmBRV1 shared 87% sequence identity with the 5′-terminus of SsBRV1 (Figures S1 and S3). The genome organizations of BmBRV1-BdEW220 and BmBRV1 were consistent with BpRV1, SsBRV1, and SsBRV2, but differed from ABRV1 [11,12,13,14,15,16]. BmBRV1-BdEW220-dsRNA1, BmBRV1-dsRNA1, SsBRV1-dsRNA1, and SsBRV1-dsRNA1 encode a cap-pol fusion protein containing an RdRp domain [11,12,13,16]. However, in ABRV1, the cap-pol fusion protein is encoded by dsRNA2 [14]. The conserved terminal sequences of viral genomic RNA are generally involved in virus packaging [49,50]. A long 5′-UTR and a relatively short 3′-UTR were highly conserved among the dsRNA segments of the members of the family *Botybirnaviridae* [11,12,13,14,16]; for instance, approximately 500 bp at the 5′-UTRs and 100 bp at the 3′-UTRs of BmBRV1-BdEW220 (Figure 3A). This type of long and strictly conserved 5′- has not been observed in other known dsRNA viruses, except for botybirnaviruses [1,11,12,13,14,15,16]. Conversely, the first start codons (ATG) of ORF1 and ORF2 are located outside the strictly conserved region of the 5′ terminal of BdBRV1, BmBRV1, and SsBRV1 [12,16], whereas those of BpRV1, SsBRV2, and ABRV1 are situated within the highly conserved region of the 5′-UTR [11,13,14].

Members of the family *Botybirnaviridae* consist of isometric viral particles of 35–38 nm in diameter [11,12,13,14]. Our results of the viral particles of BmBRV1-BdEW220 (37 nm in diameter) generally corroborated previous morphometric studies [11,12,13,14]. The structural proteins might be derived from two ORFs encoded by dsRNA1 and dsRNA2 in the family *Botybirnaviridae* [11,12,14]. The three distinct protein bands of BmBRV1-BdEW220 were 110 kDa, 90 kDa, and 80 kDa. The protein p110 was encoded by the ORF2 of dsRNA2, and p90 and p80 were encoded by the ORF1 of dsRNA1 (Figure S5). Hence, the structural proteins of BmBRV1-BdEW220 were derived from two ORFs encoded by dsRNA1 and dsRNA2. This arrangement is also observed in members of the family *Botybirnaviridae* [11,12,14]. In SsBRV1, the molecular weights of three structural proteins were 120 kDa (P120), 100 kDa (P100), and 80 kDa (P80) [12]. P80 and P100 were encoded by ORF I (dsRNA1), and P120 was encoded by the smaller fragment dsRNA2 (Figure S5) [12]. The three structural proteins of BpRV1 indicated sizes of 85 kDa, 80 kDa, and 70 kDa [11]. PMF-MS analysis revealed that the 80 and 85 kDa structural proteins of BpRV1 were encoded by ORF I (dsRNA1), while the 70 kDa structural protein was encoded by ORF II (dsRNA2) (Figure S5) [11]. The three structural proteins of ABRV1 exhibited sizes of 80 kDa, 70 kDa, and 60 kDa [14]. The isometric spherical particles of ABRV1 were putatively composed of three structural proteins encoded by ORF1 (p60) and ORF2 (p70 and p80) (Figure S5) [14]. In contrast, SsBRV2 comprised four structural protein components, with sizes of 100 kDa, 90 kDa, 70 kDa, and 60 kDa, but the ORFs that encoded them were not arranged [13]. The structural proteins of the other three botybirnaviruses were not described [14,15,16]. It is worth exploring the mechanism associated with the different sizes of the structural proteins in the family *Botybirnaviridae*. We also found a serine-rich region profile in BmBRV1-BdEW220-ORF2. In other botybirnaviruses, ABRV1-ORF2 contains a proline-rich region, and SsBRV1-ORF2 contains a GHBP domain (animal growth hormone receptor binding domain) [12,14]. However, these regions were not detected in SsBRV2, BmBRV1, and BpRV1 [11,13,15].

In the genus *Botybirnavirus*, BpRV1, SsBRV1, SsBRV2, ABRV1, BmBRV1, and BmBRV1-BdEW220 infect filamentous fungi [11,12,13,14,16], whereas SlaBRV1 was detected in a soybean phyllosphere via a metatranscriptomics technique [15]. Five mycoviruses in the family *Botybirnaviridae* have been found to infect phytopathogenic fungi, but only two were involved in conferring hypovirulence to their host fungi [11,12]. The results of the present study indicated that BmBRV1-BdEW220 is related to hypovirulence to *B. dothidea*. Similarly, BpRV1 and SsBRV2 could alter colony morphology and reduce the virulence of their host fungi [11,13]. Interesting, *S. sclerotiorum* strain infected with SsBRV1, which exhibits satellite-like RNA, showed hypovirulence [12]. The satellite-like RNA (dsRNA 3) of SsBRV1 might participate in modulating the virulence of the virus [12]. Moreover, the ABRV1-infected fungal strains maintained active growth and a normal colony morphology but exhibited attenuated virulence [14]. Furthermore, in addition to the significant differences between the BmBRV1-BdEW220-infected and BmBRV1-BdEW220-free strains, there were also significant differences between the virus-infected strains. This might be associated with the use of the sectored or normal region of the mycelial agar plugs for assaying the virulence. In combination, these findings suggest that BmBRV1-BdEW220 is closely associated with the hypovirulence of the phytopathogenic fungus *B. dothidea*.

## Figures and Tables

**Table 1:** Origin of the strains of *Botryosphaeria dothidea* used in this study.

Strain	Origin	BmBRV1-BdEW220
EW220	*Pyrus pyrifolia*, Wuhan, China	positive
JNT1111	*P. bretschneideri*, Shanxi, China	negative
EW220-64	A single-conidium isolate of EW220	negative
EW220-64-T1	EW220-64 in a pairing culture of EW220-64 and EW220	positive
EW220-64-T2	EW220-64 in a pairing culture of EW220-64 and EW220	positive
EW220-64-T3	EW220-64 in a pairing culture of EW220-64 and EW220	positive

## Supplementary Materials

The following are available online at https://www.mdpi.com/1999-4915/11/3/266/s1. Figure S1. Multiple-sequence alignments of the dsRNA1 segment of BmBRV1-BdEW220, BmBRV1 and SsBRV1. Abbreviations: BmBRV1-BdEW220, Bipolaris maydis botybirnavirus 1 strain BdEW220; BmBRV1, Bipolaris maydis botybirnavirus 1; SsBRV1, Sclerotinia sclerotiorum botybirnavirus. Figure S2: Multiple-sequence alignments of the sequences of P1 encoded by BmBRV1-BdEW220, BmBRV1 and SsBRV1. Abbreviations: BmBRV1-BdEW220, Bipolaris maydis botybirnavirus 1 strain BdEW220; BmBRV1, Bipolaris maydis botybirnavirus 1; SsBRV1, Sclerotinia sclerotiorum botybirnavirus. Figure S3: Multiple-sequence alignments of the dsRNA2 segment of BmBRV1-BdEW220, BmBRV1 and SsBRV1. Abbreviations: BmBRV1-BdEW220, Bipolaris maydis botybirnavirus 1 strain BdEW220; BmBRV1, Bipolaris maydis botybirnavirus 1; SsBRV1, Sclerotinia sclerotiorum botybirnavirus. Figure S4: Multiple-sequence alignments of the sequences of P2 encoded by BmBRV1-BdEW220, BmBRV1 and SsBRV1. Abbreviations: BmBRV1-BdEW220, Bipolaris maydis botybirnavirus 1 strain BdEW220; BmBRV1, Bipolaris maydis botybirnavirus 1; SsBRV1, Sclerotinia sclerotiorum botybirnavirus. Figure S5: Genomic organization schematic diagrams of Botrytis porri botybirnavirus 1 (BpRV1), Bipolaris maydis botybirnavirus 1 strain BdEW220 (BmBRV1-BdEW220), Sclerotinia sclerotiorum botybirnavirus 1 (SsBRV1), and Alternaria botybirnavirus 1 (ABRV1). Table S1: PMF-MS analysis of p110 encoded by ORF2 of Bipolaris maydis botybirnavirus 1 strain BdEW220. Table S2: PMF-MS analysis of p90 encoded by ORF1 of Bipolaris maydis botybirnavirus 1 strain BdEW220. Table S3: PMF-MS analysis of p80 encoded by ORF1 of Bipolaris maydis botybirnavirus 1 strain BdEW220.
