# Supplementary material for: Characterization of a Botybirnavirus Conferring Hypovirulence in the Phytopathogenic Fungus Botryosphaeria dothidea
Source: Viruses. 2019 Mar 17;11(3):266. doi: 10.3390/v11030266 (PMC6466033; doi:10.3390/v11030266)
Supplement: Supplementary file 1 [file viruses-11-00266-s001.zip › viruses-449497-supplementary/Manuscript supplementary Figure 1.docx]

**Supplementary**

**Figure S1.** Multiple-sequence alignments of the dsRNA1 segment of BmBRV1-BdEW220, BmBRV1 and SsBRV1. Abbreviations: BmBRV1-BdEW220, Bipolaris maydis botybirnavirus 1 strain BdEW220; BmBRV1, Bipolaris maydis botybirnavirus 1; SsBRV1, Sclerotinia sclerotiorum botybirnavirus.

BmBRV1-BdEW220 1 GCAATAAAAGGCGAAGCCGGGAGGCTTCTATTTTATTGCCCAGTTTTTCATCTGGTCAAA

BmBRV1 1 GCAATAAAAGGCGAAGCCGGGAGGCTTCTATTTTATTGCCCAGTTTTTCATCTGGTCAAA

SsBRV1 1 GCAATAAAAAGCACAGCCGGAAGGCTTTCTTTTTATTGCCCAGTTTTTGATCTGGTTAAA

BmBRV1-BdEW220 61 ATGAAGTCGGCTATC--TAGCCAGCGGAGAGTGGAACTGGTCCTCTTCGTACGAAACCAA

BmBRV1 61 ATGAAGTCGGCTATC--TAGCTAGCGGAGAGTGGAACTGGTCCTCTTCGTACGAAACCAA

SsBRV1 61 ATCAAACTGGCTTGCCATAGCCACCAGAAGATGGAACTGGTCTCTTCTGTTCTACACCAA

BmBRV1-BdEW220 119 ACCGAGCTCCCACCCGGTGAGGTAATCCAGTGGCAATGCGTGGGGGAGACCTCGGCCT--

BmBRV1 119 ACCGAGCCCCCACCCGGTGAGGTAATCCAGTGGCAATGCGTGGGGGAGACCTCGGCCT--

SsBRV1 121 TCCGAGCTCCCACCCGGTGCGGTAATCCAGTGGTAATGCGTGGGGGAGACCTCGGTTTTA

BmBRV1-BdEW220 177 ---------CGGCCGAGTGATTGTGCAGAACGGGTGGTGATGTCAGCAATGCTCACCGTA

BmBRV1 177 ---------CGGCCGAGTGATTGTGCAGAACGGGTGGTGATGTCAGCAATGCTCACCGTA

SsBRV1 181 AGCTTAAAGCAGCCGGGTGATTGTGCAGAACGGGTGGTGATGTCAGCAATGCTCACCGTA

BmBRV1-BdEW220 228 CATGTCCGTGCTAGGGAGAATGTCCTGTATCCAGCAGGAAGATACCCCGCGTGTAACGAC

BmBRV1 228 CATGTCCGTGCTAGGGAGAATGTCCTGTATCCAGCAGGAAGATATCCCGCGTGTAACGAC

SsBRV1 241 CATGTCCGTGCTAGGGGGAATGTCCTGTATCCAGCAGGAAGATACCCCGCGTGTAACGAC

BmBRV1-BdEW220 288 TGCAAGCACTTTGTCTCCTACGTAGAACAGTCTATCACCTAGCGGCGCTGATCGGCCGAT

BmBRV1 288 TGCAAGCACTTTGTCTCCTACGTAGAACAGTCTATCACCTAGCGGCGCTGATCGGCCGAT

SsBRV1 301 TGCAAGCACTTTGTCTCCTACGTAGAACAGTCTACCACCTAGCGGCGCTGATCGGCCGAT

BmBRV1-BdEW220 348 ACAGTAGGTGGTGTGTAGGCTTGCC-AGCCCAACCCCCGCCACAGACATAGCTTCCAAAC

BmBRV1 348 ACAGTAGGTGGTGTGTAGGCTTGCC-AGCCCAACCCCCGCCACAGACATAGCTTCCAAAC

SsBRV1 361 ACAGTAGGTGGTGTGTAGGCTTACCAAACCCGACCCACGCCACAG-CAAAGTTACCAAGC

BmBRV1-BdEW220 407 AACTACACACACACAATATAGCAGAGTGAGCTAATCCAGCAGCTATATT-AACACACTAC

BmBRV1 407 AATTACACACACACAATATAGCAGAGTGAGCTAATCCAGCAGCTATATT-AACACACTAC

SsBRV1 420 CA--ACACAAATACAATATAGTAGAGTGAGCTAATCCAGCAACTATATTGAACAATCTAC

BmBRV1-BdEW220 466 AGTAGATCAACTTTTGAATACTGTTATCGATC-------------ACACACAAGATTCTA

BmBRV1 466 AGTAGATCAACTTTTGAATACTGTTATCGATC-------------AAACACAAGAATCCA

SsBRV1 478 AGTAGATCAACTTTTGAATACTGTTTTCGATCAACATACCAAATAACACAACAAAATACA

BmBRV1-BdEW220 513 TATACCAATCTCACAAATTACAAAACACAAAAA-ATGTCTTTGAACTTTGCTTCTATTTC

BmBRV1 513 TATACCAATCTCACAAATTACAAAACACAAAAAAATGTCTTTGAACTTTGCTTCTATTTC

SsBRV1 538 AAAACAAAT----TAAATTTAAAAACCCAAAAATATGTCTTTTTCAAACAACATCGTTTC

BmBRV1-BdEW220 572 TGATTTTGCTTCTGCTGCCTCCATTGCTTTACCGCTTGAGATGTGGATTGAGATTGTCGC

BmBRV1 573 TGATTTTGCTTCTGCTGCCTCCATTGCTTTACCGCTTGAGATGTGGATTGAGATTGTCGC

SsBRV1 594 TGTTTCTGCTTTACCTACCCTGGCTACGTTGCCGTACGAGATGTGGAGCAGCGTTGTGTC

BmBRV1-BdEW220 632 GAATTTGAGCACATGGGATGCTGCTTGTATCGTCAAGAACATCCCTGTGTGCAGAGCGCC

BmBRV1 633 GAATTTGAGCACATGGGATGCTGCTTGTATCGTCAAGAACATCCCTGTGTGCAGAGCGCC

SsBRV1 654 TAACCTGAGCACATGGGATGCCGCTTGCATCGTCAAGAACATCCCAATGTGCCAGTCTTT

BmBRV1-BdEW220 692 GGTGTACACAGCCTTATTGGCTCGTGCAAACCAGGAGTTCAATTTCGGGTGCTGCGTTGA

BmBRV1 693 GGTGTACACAGCCTTATTGGCTCGTGCAAACCAGGGGTTCAATTTCGGGTGCTGCGTTGA

SsBRV1 714 GTTGTACCAAGCACTCTTAGCCCGAGCTAACCTTGACTACAACTTCGGTTGCACCCATGA

BmBRV1-BdEW220 752 AGAAATCATTTCTTCAATTCCTTGCTCCCACTCCCGTCCTTGCGAAATTGTTTCCAAGGA

BmBRV1 753 AGAAATTATTTCTTCAATTCCTTGCTCCCACTCCCGTCCTTGCGAAATTGTTTCCAAGGA

SsBRV1 774 GAAGATCATCTCTACTATGCCGTGTAACCACCCCCGCACATGCGAGGTGCTCCCGAAAGG

BmBRV1-BdEW220 812 TGCCCAATTCTCGCTCGCGTGCGTAGAGCACATTAGTGCAAGCTGCGGATCGAGCTGCGA

BmBRV1 813 TGCCCAATTCTCGCTCGCGTGCGTAGAGCACATTAGTGCAAGCTGCGGATCGAGCTGCGA

SsBRV1 834 CGGCCACTTCGCATTCGTATGTGCTGAGCACATCGTGGGTACATGCAGCGCTAGTTGCGA

BmBRV1-BdEW220 872 GAAAGTGCTCATTAGTGAGTACGATTTAGGAGTGCGGCTCAAGCACCGCGAAGCTGAACG

BmBRV1 873 GAAAGTGCTCATTAGTGGGTACGATTTAGGAGTGCGGCGCAAGCACCGCGAAGCTGAACG

SsBRV1 894 TAGTGTGCTCATGAGTGCCGATGACGTGTACTCACGTTTTAAGCGCTACGAAGAGTCACG

BmBRV1-BdEW220 932 TGCCCAGCGAAGATTGGACCGTATTTACGGCCCACTCGCTACTGAGCACGACATGATGGT

BmBRV1 933 TGCCCAGCGAAGATTGGACCGTATTTACGGCCCACTCGCTACTGAGCACGACATGATGGT

SsBRV1 954 GCTCCAACGCAAGCGCGACCATATGTATGGTCCATTTGCGTTGGAGCATGACATGATGGT

BmBRV1-BdEW220 992 CATTGATGACCAACACTCCGAGACTTCTTCCTATAGAGATTATCTTCGCTACAGCGCAGA

BmBRV1 993 CATTGATGACCAACACTCCGAGACTTCTTCCTATAGAGATTATCTTCGCTACGGCGCAGA

SsBRV1 1014 CATCGATGACCAAAACGCCGCGACTTCATCGCACCGAGACTACATGCGCTACCGCGCAGA

BmBRV1-BdEW220 1052 GATCGCTTACACGGAAGAAGATCTCCATATCTCTTCACTCTTCGAAGCTGTCAGCTTCCA

BmBRV1 1053 GATCGCTTACACGGAAGAAGATCTCCATATCTCTTCACTCTTCGAAGCTGTCAGCTTCCA

SsBRV1 1074 GATGGTCTATGTGGAAGAAGATCTCGCACTGGGCACACTGTTTGGAGGCGATCTTGCCTC

BmBRV1-BdEW220 1112 ACCAGAGGGTAGTGATGTAGAGAAGACTCGGGGTGGTATGGATGCTGCCGATGCTGGTCC

BmBRV1 1113 ACCAGAGGGTAGTGATGTAGAGAAGACTCGGGGTGGTATGGATGCTGCCGATGCTGGTCC

SsBRV1 1134 TACAGACACCCTAGTTGTGGAGAGAACTCGTGGTGGTATGGATGCTGCAGGCTCTGGTGC

BmBRV1-BdEW220 1172 TTCTGAATCAGCACAGGCTGCTCCTACACCCGTAGATGATGAGGTCCCTCAAGACGGACC

BmBRV1 1173 TTCTGAATCAGCACAGGCTGCTCCTACACCCGTAGATGATGAGGTCCCTCAAGACGGACC

SsBRV1 1194 TCCAGAAGCATCAGCTGCAGCCCCTACGCCCGTCGAAGACGAGGTACCACAAGATGGGCC

BmBRV1-BdEW220 1232 TGTCACCGGAGAGCCAACCGTTGGGCTGCCACCAGGAAGTGAGGAGCCGGGCCACCCAGG

BmBRV1 1233 TGTCACTGGAGAGCCAACCGTTGGGCTGCCACCAGGAAGTGGGGAGCCGGGCCACCCAGG

SsBRV1 1254 TGTCAACGAAGAGCCCGTCGTCGGGCTACCCCCCGGAAGTGAAGAACCGAGCCATCCAGG

BmBRV1-BdEW220 1292 CCCAGACGTCCCTCTTGACCCTAGGGTAACTAATGGCGATGACTTTGGTCATATTCCAAA

BmBRV1 1293 CCCAGACGTCCCTCTTGACCCTAGGGTAACTAATGGCGATGACTTTGGTCATATTCCAAA

SsBRV1 1314 ACCCGATGTTCCACTCGACCCGAGGGTCACCAACGGCGATGATTTTGGTAACATTCCAAA

BmBRV1-BdEW220 1352 CACCGAAGATGCATCCGACCCAGTCGATGCAGTCGCCACAAACGGTTGGTTTGAATACCC

BmBRV1 1353 CACCGAAGATGCGTCCGACCCAGTCGATGCAGTCGCCACAAACGGTTGGTTTGAATACCC

SsBRV1 1374 CACGGAAGATGCATCTGACCCTGTCGATGCTGTTGCCACAAACGGTTGGTTTGAGTACCC

BmBRV1-BdEW220 1412 ATACGCCACAGGCGGTGGGCAGGCTACATCATACGCAGGCCTGATCACACAAGCAGACCC

BmBRV1 1413 ATACGCCACAGGCGGTGGGCAGACTACATCATACGCAGGCCTGATCACACAAGCAGACCC

SsBRV1 1434 CTATGCCGAAGGCAGCGGGCAAGTCGCATCTTATGCAGGCTTGATTACCCAAGCCAACCC

BmBRV1-BdEW220 1472 CACTTCTGAGTATGTTACAAATATCTCAGACCGATTCCGAAGCATCTCTCCTCAGTTTGA

BmBRV1 1473 CACTTCTGAGTATGTTACAAATATCTCAGACCGATTCCGAAGCATCTCTCCTCAGTTTGA

SsBRV1 1494 ATCCTCTGAGTATGTTACCAACATCTCAGACCGATTCCGCAACATCTCTCCTCAATTTGA

BmBRV1-BdEW220 1532 GGAACTTGAGCGTAACATTCGCGTTACTTCAGGATTCGGTGTCCAGTCTTACTTGGTGCA

BmBRV1 1533 GGAACTTGAGCGTAACATTCGCGTTACTTCAGGATTCGGTGTCCAGTCTTACTTGGTGCA

SsBRV1 1554 GGAGTTAGAGCGCAATATTCGCGTCACCTCCGGAAGCGGCGTACAGTCGTACTTGGTGCA

BmBRV1-BdEW220 1592 GACACTCTGGGGCTTTGGACCCCGCGGTGCTTCATCACTGGTGACGCAATCGGCAAATGC

BmBRV1 1593 GACACTCTGGGGCTTTGGACCCCGCGGTGCTTCATCACTGGTGACGCAATCGGCAAATGC

SsBRV1 1614 GACTATCTGGGGCTTTGGTCCTAGAGGTGCTGCCTCACTTATGACGCAGTCAGCAAATGC

BmBRV1-BdEW220 1652 TGATTCTCGGACTGTAGCTTTTTGGACTACGAACCCCAAATCTGAGATCATACCCGTGAG

BmBRV1 1653 TGATTCTCGGACTGTAGCTTTTTGGACTACGAACCCCAAATCTGAGATCATACCCGTGAG

SsBRV1 1674 TGATACTCGGACTGTAGCCTTTTGGACTACAAACCCCCAGTTCGAGATTGTCCCTGTAAG

BmBRV1-BdEW220 1712 TGAGGATACTATTATGTATGACACACTTGCCGGGCTGTCTATTGAAGGACAGATGGTAAG

BmBRV1 1713 TGAGGATACTATCATGTATGACACACTTGCCGGGCTGTCTATAGAAGGACAGATGGTAAG

SsBRV1 1734 CGAAGATACGATCATGTACGACACGCTCGCAGGACTATCTGTTGAAGGTCAAATGGTTCG

BmBRV1-BdEW220 1772 ACTGAATACTACCTTCAACGGCAACATGGTTACCGATCTATATAATAGTATCGGTGACAG

BmBRV1 1773 GCTGAATACTACCTTCAACGGCAACATGGTTACCGATCTATATAATAGTATCGGTGACAG

SsBRV1 1794 CTTGAACACAACCTTCAACGATAACATGATCACTGACCTGTACAACAGTGTCGGCGACCG

BmBRV1-BdEW220 1832 GGCTATCGCTGAGCGGTACTACGACCACGTAGTGACCGCTGCAGTGGCAGGAAGTAACTA

BmBRV1 1833 GGCTATCGCTGAGCGATACTACGACCACGTAGTGACCGCTGCAGTGGCAGGAAGTAACTA

SsBRV1 1854 TGCCATCGCACAGCGTTACTACGACCACGTAGTAACAGCAGCAGTAGCTGGAAGTAATTA

BmBRV1-BdEW220 1892 TGTTGCTTTTCTGACCATGTGCTACACGCGATTGGTCTCACTCAAGATCATGGCTGAGCA

BmBRV1 1893 TGTTGCTTTTCTGACCATGTGCTACACGCGATTGGTCTCACTCAAGATCATGGCTGAGCA

SsBRV1 1914 CGTTGCTTTCTTGACAATCTGCTATACGAGGCTCGTCGCGCTGAAGATCATGTCTGAGCA

BmBRV1-BdEW220 1952 AAATCAGCCGGCCACAATGCGCGTTGAGGGTGACACTCTCAACGCCAACATTTTACTTGA

BmBRV1 1953 AAATCAACCGGCCACAATGCGCGTTGAGGGTGACACTCTCAACGCCAACATTTTACTGGA

SsBRV1 1974 GAACCAGCCTGCCCAGATGCGCGTTTCAGGAGATACACTGAACGCAAATATCTTGCTGGA

BmBRV1-BdEW220 2012 TAACGTTGTAGCGAACTCAGTTACCAGAAGGGTAGCGGATGCCGTATTGCGTTCCAAGCC

BmBRV1 2013 TAACGTTGCAGCGAACTCAGTTACCAGAAGGGTAGCGGATGCCGTATTGCGTTCCAAGCC

SsBRV1 2034 TACCGTTGCTGCTAATCCTGTAACTCGGAGAGTAGCCGATGCAGTGTTGCGCTCAAAGCC

BmBRV1-BdEW220 2072 CACCAATGCTGTCATGCTCCCACATGGCAGCAACGACCTTGATGTCGAGACCATGCTCTA

BmBRV1 2073 CACCAATGCTGTCATGCTCCCACATGGCAGCAACGACCTTGATGTCGAGACCATGCTCTA

SsBRV1 2094 AACGAATGCAGTCATGCTCCCACATGGCAGCAACGATCTCGATGTCGAGACCATGCTTTA

BmBRV1-BdEW220 2132 CTTGATGGGGCACGGCCGCGTTATCAAGGGCGTCGCAACCGAAGATGAAGAGATAGCGGT

BmBRV1 2133 CTTGATGGGGCACGGCCGCGTTATCAAGGGCGTCGCAACCGAAGATGAAGAGATAGCGGT

SsBRV1 2154 TTTGATGGGCCATGGACGCGTTATCAAGAGTGTCGCTACAGATGAAGACGAAGTTGCTGT

BmBRV1-BdEW220 2192 CTTTTCACCTTTCGACCGATTCCATACCGACTCAAACTTCAAGCTGGTAGGCTTGGTTGG

BmBRV1 2193 CTTTTCACCTTTCGACCGATTCCATACCGACTCAAACTTCAAGCTGGTAGGCTTGGTTGG

SsBRV1 2214 TTTTTCACCATTTGACCGGTTCCACACAGACTCCAACTTTAAGTTGTTAGGAGTTGTTGG

BmBRV1-BdEW220 2252 GGAGAATGCGATTGGACACTTCCCAGCTGGGAATTTAGAATTCCAAATCGACTTCGGCCA

BmBRV1 2253 GGAGAATGCGATTGGACACTTCCCAGCTGGGAATTTAGAATTCCAAATCGACTTCGGCCA

SsBRV1 2274 GGAGCGAGCAATTGGCAATTTCCCTCCCGGGAACTTGGAATTCCAGATCGACTTCGGCCA

BmBRV1-BdEW220 2312 AGCGTTCGATTTGTTGAACCGCTATGTAAATCAGAATGACTTATGGGATCAGTTTGCCAT

BmBRV1 2313 AGCGTTCGTTTTGTTGAACCGCTATGTAAATCAGAATGATTTATGGGATCAGTTTGCCAT

SsBRV1 2334 AGCGTTTGACCTCCTGAACCGCTACATCAACCAAAATGATTTGTGGGATCAGTTTGCCAT

BmBRV1-BdEW220 2372 TGCACGCAACATTGCACTCGGTATGATCTTCTCCAGATCATTCTCAGCTTCAGTAGGGCT

BmBRV1 2373 TGCACGCAACATTGCACTCGGTATGATCTTCTCCAGATCATTCTCCGCTTCAGTAGGGCT

SsBRV1 2394 TGCACGAAACATTGCTTTGGGGATGATCTTCTCACGAACATTCTCTTCCTCGGTTGGGTT

BmBRV1-BdEW220 2432 TCCCAAGCCTTACCACTCACGCGATCTTGCTCTGAACAGAACAAGTACCGGCGCTAACCA

BmBRV1 2433 TCCCAAGCCTTACCACTCACGCGATCTTGCTCTGAACAGAACAAGTACCGGCGCTAACCA

SsBRV1 2454 GCCAAAACCCTACCATTCACGGGACCTTGCACTCAATAGGACCCATACTGGCGCCAACCA

BmBRV1-BdEW220 2492 GCATGGCAGGCGGCGGGTTATGGAATTCAAGGAGTCTTTCCATGCTTTAGTAGCCTCAGG

BmBRV1 2493 GCATGGCAGGCGGCGGGTTATGGAATTCAAGGAGTCTTTCCATGCTCTAGTAGTCTCAGG

SsBRV1 2514 ACATGGTAGGCGCCGCGTGATGGAGTTCAAAGAGTCCTTCCACGCTGTTGTTGCCTCGGG

BmBRV1-BdEW220 2552 AACGTGGCACTGCGCAGCAATGGAAGAGACACTGTTTGAGTCCGTCGTGAACGTAATGGA

BmBRV1 2553 AACGTGGCATTGCGCAGCAATGGAAGAGACACTGTTTGAGTCCGTCGTGAACGTAATGGA

SsBRV1 2574 AACTTGGCATTGTGCAGCTATGGAGGAGACACTCTTTGAGTCAGTAGTCAACGTGATGGA

BmBRV1-BdEW220 2612 AGAGACAGCTGGCATAGGTCCCCGTGCCCCGAACTTCTATGCAACAATTGATACGATGCA

BmBRV1 2613 AGAGACAGCTGGCATTGGTCCCCGTGCCCCGAACTTCTATGCAACAATTGATACGATGCA

SsBRV1 2634 AGAGACAGCTGGTATCGGACCACGAGCACCAAATTTCTATGCGACGATTGATACGATGCA

BmBRV1-BdEW220 2672 AGATGATTTTGATCTTGAGTACAAGACAGCAATGGTCTGCCTCCCGGTAGTCGAACGCAT

BmBRV1 2673 AGATGATTTTGATCTTGAGTACAAGACAGCAATGGTCTGCCTCCCGGTAGTCGAACGCAT

SsBRV1 2694 AGATGACTTCGACCTTGAGTACAAAACTGCGATGGTCTGCCTTCCGGTAGTCGAGCGCAT

BmBRV1-BdEW220 2732 GACTGGTACTCCTTCGAACCATATCCACCAGTATGTTAGCAAGAGCAACACTGCCTTTAT

BmBRV1 2733 GACTGGTACTCCTTCGAACCATATCCACCAGTATGTTAGCAAGAGCAACACTGCCTTTAT

SsBRV1 2754 GACAGGTACTTCGTCGAGCCACATCCACCAATATGTCAGCAAGAGCAATGTTGCTTTCAT

BmBRV1-BdEW220 2792 GAAGGCTATCAGCTTTGGATGGGAATCGAAGCCAATCCGCATCTCCAGCTACTTAGCTTT

BmBRV1 2793 GAAGGCTATCAGCTTTGGATGGGAATCGAAGCCAATCCGCATCTCCAGCTACTTAGCTTT

SsBRV1 2814 GAAAGCCATCAGTTTTGGTTGGGAGTCGAAACCTATTCGCATTTCCAGCTACCTCGCTCT

BmBRV1-BdEW220 2852 GGAGATCACTCCTGAAGATAAGAATTTCAAATTCTTGTTCGACAAGAGTGCCCGTACTGC

BmBRV1 2853 GGAGATCACTCCTGAAGATAAGAATTTCAAATTCTTGTTCGACAAGAGTGCCCGTACTGC

SsBRV1 2874 CGAAATTACCCCCGAAGATAAAAATTTCAAGTTCTTATTTGACAAGAGTGCACGAACAGC

BmBRV1-BdEW220 2912 ACTCCTCAAACAAGAGAAGTACACATGGCGAGAGTCTGTGCTGACCTCGTTTGTCTGCCA

BmBRV1 2913 ACTCCTCAAACAAGAGAAGTACACATGGCGAGAGTCTGTGCTGACCTCGTTTGTCTGCCA

SsBRV1 2934 GCTGCTCAAGCAGGAGAAGTTTACATGGCGTGAAGCTGTGTTGACTTCTTTCGTCTGCCA

BmBRV1-BdEW220 2972 CTCACAGTGGGGACCCAGCGAGCAGCACCGGTCTTACGGTGAGTTTTACGATGATGGCAT

BmBRV1 2973 CTCACAGTGGGGACCCAGCGAGCAGCACCGGTTTTACGGTGAGTTTTACGATGATGGCAT

SsBRV1 2994 CTCGCAGTGGGGTCCCAGTGAACAGCACCGATTCTACGGTGAGTTCTATGATGATAATAT

BmBRV1-BdEW220 3032 CTCTGCCCTCCGAGAGCAAACCCCTTTCTACGGGTATCTCGGTAGAAGTCAACTCGAACA

BmBRV1 3033 CTCTGCCCTCCGAGAGCAAACCCCTTTCTACGGGTATCTCGGTAGAAGTCAACTCGAACA

SsBRV1 3054 CTCTGACCTCCGCGAACAGGTTCCCTTCTATGGGTATCTAGGCCGAAGCCAGCTTGAACA

BmBRV1-BdEW220 3092 CTTGGAAGGTACGCCTACCATTTGGAACACGAGCACAGCTGCTACACGAATTGAGTTCAC

BmBRV1 3093 CTTGGAAGGTACGCCTACCATTTGGAACACGAGCACAGCTGCTACACGAATTGAGTTCAC

SsBRV1 3114 TCTCGAAGGTTCACCGACCATTTGGAATACTAGCACCGCTGCTACACCTCTTGAGTTCAC

BmBRV1-BdEW220 3152 TAAGCCAGCTAGCCTGGCCCGCGCAACGCATGGGCTCAACGAAGATGAAGCTTCTCAGGT

BmBRV1 3153 TAAGCCAGCTAGCCTGGCCCGCGCAACGCATGGGCTCAACGAAGATGAAGCTTCTCAGGT

SsBRV1 3174 AAAACCGAAGAGCTTGACACGAGTCACTCGTGATCTCAACGAAGAGGAGGCCATGCAAGT

BmBRV1-BdEW220 3212 AGACTCACTTTGGGCTACTATGAAAGCAGCACTTGAGGCTGCTTCTGCAGACAACGATGC

BmBRV1 3213 AGACTCACTTTGGGCTACTATGAAAGCAGCACTTGAGGCTGCTTCTGCAGACAACGATGC

SsBRV1 3234 GGACTCTTTGTGGGCCACCATGAAAGCCGCACTTGAAGCTGCCTCTGCAGAGAATGATGC

BmBRV1-BdEW220 3272 TGAGGAGAGTGACGATGATGAATTTGTCAGCCCTCCGCAACCAACTGCAAACCCAACGCA

BmBRV1 3273 TGAGGAGAGTGACGATGATGAATCTGTCAGCCCTCCGCAACCAACTGCAAACCCAACGCA

SsBRV1 3294 CGAGGAAGATGAAGATGATGAGGAATTTGAGTACCCACAACCAACTGCTAACCCAACAGG

BmBRV1-BdEW220 3332 AAAGGGCCGGCGCTTCGAAGTCCGCGAGCCTAAAGGCAAGGAGATAGCCCGGGTTGTACC

BmBRV1 3333 AAAGGGCCGGCGCTTCGAAGTCCGCGAGCCTAAAGGCAAGGAGATAGCCCGGGTTGTACC

SsBRV1 3354 CCAGGGACAGCGCTTCGAGCTCCACGAGCCGAAAGGTAAGGAGGTAGAGCGCAGTATACC

BmBRV1-BdEW220 3392 TGAACCAGAACACCAGCGCGCTGGCACAGACAGGCCGATCCCCACTTCGCAGTGGCAAAA

BmBRV1 3393 TGAACCAGAACACCAGCGCGCTGGCACAGACAGGCCGATCCCCACTTCGCAGTGGCAAAA

SsBRV1 3414 TGAGCCGGAACAGCAGCGTGCTGGCACTGATCGCCCGATCCCTGCTTCGCAGTGGCAAAG

BmBRV1-BdEW220 3452 ACCTAAGCATACTGCTAAGGCTGTAACTGCTGCTAATCAAAATGGGTTAGTCCAAA---A

BmBRV1 3453 ACCTAAGCATACTGCTAAGGCTGTAATTGCTGCTAATCAAAATGGGTTAGTCCAAA---A

SsBRV1 3474 ACCTAAGAATGCAGCCCG---TGTCAGTGCAAGTGTCGAGAAGACACAAGTACAGGCCCG

BmBRV1-BdEW220 3509 GAACTATTTCCAAAAGTTGACTCCAGCTCCTACTTTGATTGGGCTGAACCCCAACGGAGG

BmBRV1 3510 GAACTATTTCCAAAAGTTGACTCCAGCTCCTACTTTGATTGGGCTGAATCCCAACGGAGG

SsBRV1 3531 GAATTACTTCCAAGGTTTAGCAACTCCAAGTAGGTTGATCGGTTTCAATCAAAGCACACC

BmBRV1-BdEW220 3569 CACCCCAGGTGCACAGGCCCAAGGCCCGAGTAGGCAGCTGACTACAAGTACCATCAAGCG

BmBRV1 3570 CACCCCAGGTGCACAGGCCCAAGGCCCGAGTAGGCAGCTGACTACAAGTACCATCAAGCG

SsBRV1 3591 GACACCTGAAATGGTGCCACAACAGCGGAACTCTCCCCTCGCGATCAAGACTCTTAAGAA

BmBRV1-BdEW220 3629 GATGACCCCAAAGCAATCCGATCGCGCAGATCATGCCTACTTGGACTCACAAATCAAGAG

BmBRV1 3630 GATGACCCCAAAGCAATCCGATCGCGCAGATCATGCCTACTTGGACTCACAAATCAAGAG

SsBRV1 3651 GGTCTCTGATCGCCAAAAGGACTACGAAGAGCATGTATACTTGGATACCCAAGTCAAGAA

BmBRV1-BdEW220 3689 AGTCACGTCTGAGCGTCGTCTGTTTGAGCAAGCTAACCGCGGTGGT------------GC

BmBRV1 3690 AGTCACGTCTGAGCGTCGTCTGTTTGAGCAAGCTAACCGCGGTGGT------------GC

SsBRV1 3711 AACAGCCGCCGAACGTAGGCAGTTCGAGCAATACCATCGAGGTGGTATGCAGAAGAAAGC

BmBRV1-BdEW220 3737 TACTCGCTCTGGCTCTAAGAAGAAAGAGAAGGTGATCCCCGCCGTACTCCGTGACGAGGT

BmBRV1 3738 TACTCGCTCTGGCTCTAAGAAGAAAGAGAAGGTGATCCCCGCCGTACTCCGTGACGAAGT

SsBRV1 3771 AAATCGC------------AAGGATAAGAGAGTCGCCCCTGCACAAGTTCGCGATGAGGT

BmBRV1-BdEW220 3797 CGAGCAACTGTGTTCTCGCATGGTAACTGACCTCAGGTTCCGTGTAGACCTACTGACTAG

BmBRV1 3798 CGAGCAACTGTGCTCTCGCATGGTAACTGACCTCAGGTTCCGTGTGGACCTACTGACTAG

SsBRV1 3819 AGAACAACTATGCTCACGCATGGTCACTGATCTGAGGTTCAGAACCGAGCTCCTCACAAG

BmBRV1-BdEW220 3857 ACTCCCATCAACTGAAGACGAATCTAAAGCTGTCGACTTCTTGTATCCCAGGGGGAAAGA

BmBRV1 3858 ACTCCCATCAACTGAAGACGAATCTAAAGCTGTCGACTTCTTGTATCCCAGGGGAAAAGA

SsBRV1 3879 ACTACCAGCCACCGAGAATGAGTCTAAGGCCGTAGATTTCCTGTACCCGAGGGGAAAAGA

BmBRV1-BdEW220 3917 TGGCTCGCTGAAGCGAGCTGTATACACAATCGGGACACTCTTGAGGAAGCTCAAGACAGA

BmBRV1 3918 TGGCTCGCTGAAGCGAGCTGTATACACAATCGGGACACTCTTGAGGAAGCTCAAGACAGA

SsBRV1 3939 TGGATCACTCAAGCGGGCTGTCTACACAATCGGAACGCTCTTGCGAAAGCTTAGATGCGA

BmBRV1-BdEW220 3977 TAGAAAACTCACAGTTGAACAGCAGGCAGATATCAGTCTGTTCTTGAATTCCAACGTCGG

BmBRV1 3978 TAGAAAGCTCACAGTTGAACAGCAAGCAGATATCAGTCTGTTCTTGAATTCCAACGTCGG

SsBRV1 3999 TGAAAAACTCACAGCTACACAGAAGTCCGACATCAATCTTTTCCTGACAACCAACGTCGG

BmBRV1-BdEW220 4037 CGGGAAGAATGCTTGGGCTGTAGCCATAGTGATGTTTATCACGTTAAACACTCTGACACC

BmBRV1 4038 CGGGAAGAATGCTTGGGCTGTAGCTATAGTGATGTTTATCACGTTAAACACTCTGACACC

SsBRV1 4059 AGGGAAGAATGCTTGGGCTGTTGCTGTAGTCATGTTTATCTCATTGAACACACTCACCCC

BmBRV1-BdEW220 4097 AGAGTGCTACCAGATGCTCAAGAGCTACGGTCTACTCACTACCCAGTACAACCACTGGAA

BmBRV1 4098 AGAGTGCTACCAGATGCTCAAGAGCTACGGTCTACTCACTACCCAGTACAACCACTGGAA

SsBRV1 4119 AGATTGCTACGAGATGCTTAAGAGCTACGGATTCTTGACAACACCATACAACCAATGGAA

BmBRV1-BdEW220 4157 CGACAAGTGGAGCAGAATCAATGATATGTTCCGCAACCAGATGGACTCTGAGACATGGGG

BmBRV1 4158 TGACAAGTGGAGCAGAATCAATGATATGTTCCGCAACCAGATGGACTCTGAGACATGGGG

SsBRV1 4179 TGATAAGTGGAGTAAGATTAATGACTTATTCCGCAATCAAATGGATTCCGAGACATGGCT

BmBRV1-BdEW220 4217 GTTTTCTGAAACAGATTTCCCGCAATGTCTTTACATTGCTGGGTTTGTAGGGAGACCACA

BmBRV1 4218 GTTTTCTGAAACAGATTTCCCGCAATGTCTTTACATTGCTGGGTTTGTAGGGAGACCACA

SsBRV1 4239 ATACTCCGAGACTGATTTCCCGCAGTGCTTATACATTGCTGGGTTCGTAGGTCGCCCGCA

BmBRV1-BdEW220 4277 TCGTGAGGCTGATTGGGAAGCAGAGAACATCAAGCGCTCTGCAGAACCAAAGCCTATCAA

BmBRV1 4278 TCGTGAGGCTGATTGGGAAGCAGAGAACATCAAGCGCTCTGCAGAACCAAAGCCTATCAA

SsBRV1 4299 CAGGGAAGCAGACTGGGAAGCTGAGAATGCCAAAAGGTCTGCTGAACCAAAGCCAATTCG

BmBRV1-BdEW220 4337 GAAGTACACCAAGTCTGGTTTCCAAGATATGTCGGAGGAAGATGAGCGGGTGATGATCTT

BmBRV1 4338 GAAGTACACCAAGTCTGGTTTCCAAGATATGCCGGAAGAAGATGAGCGGGTGATGATCTT

SsBRV1 4359 GAAGTTTACCAAGTCAGGATTTGTAGACATGAGTGAAGAGGATGAGAAGAGAATGATCTT

BmBRV1-BdEW220 4397 AGATTTTCTTTACTCCGAAGCTTCATTCAGGATCAAGCGCGTCCAAGGATTTGAGCGGTG

BmBRV1 4398 AGATTTTCTTTACTCCGAAGCTTCATTCAGGATCAAGCGCGTCCAAGGATTTGAGCGGTG

SsBRV1 4419 GGATTTTCTATACTCAGAAGCAACATTCAGAATCAAGAGAGTGCAAGGGTTTGAACGCTG

BmBRV1-BdEW220 4457 GTACAGGAACCGCGCTGAGTGGATGATCAAAGGTTCAATGTCAGGTGAAAAAACAATCCT

BmBRV1 4458 GTACAGGAACCGCGCTGAGTGGATGATCAAAGGTTCAATGTCAGGTGAAAAAACAATCCT

SsBRV1 4479 GTACCGAAGTCGTGCAGAGTGGATGATTAAAGGATCAATGTCTGGTGAAAAGACAATCCT

BmBRV1-BdEW220 4517 TGACACAGAACCAGTAGTTATGGCAAAACTGAAGGACTTGGGTCTTAAAGTAGATGGGCA

BmBRV1 4518 TGACACAGAACCAGTAGTTATGGCAAAACTGAAGGACTTAGGTCTTAAAGTAGATGGGCA

SsBRV1 4539 TGATACAGAACCTGTTGTGATGGCAAAGCTCAAGGACTTAGGTCTGAAAGTCGATGGTCA

BmBRV1-BdEW220 4577 TGCTAATAAGATGCATATCGCTGAGAAAGTAGATTACACATGGATGATCGCTGTGCTCGA

BmBRV1 4578 TGCTAATAAGATGCATATCGCTGAGAAAGTAGATTACACATGGATGATCGCTGTGCTCGA

SsBRV1 4599 TGCAAACAAGATGCATATCGCTGAGAAAGTCGATTACACTTGGATGATTGCCGTACTAGA

BmBRV1-BdEW220 4637 CATGGACCCAGTCCATCTCGCAAAAATGCACACCAAAGGCCAAGAGAATGGTAAGGTACG

BmBRV1 4638 CATGGACCCAGTCCATCTCGCAAAAATGCACACCAAAGGCCAAGAGAATGGTAAGGTACG

SsBRV1 4659 CATGGACCCCGTCCATCTAGCTAAGATGCATACCAAAGGTCAAGAGAATGGGAAAATTCG

BmBRV1-BdEW220 4697 ATCTATCCAAGGGAGCTGCTACAGCCACTACGTCTTCGGGAATTATTGGAGTACGCATTT

BmBRV1 4698 ATCTATCCAAGGGAGCTGCTACAGCCACTACGTCTTCGGGAATTATTGGAGTACGCATTT

SsBRV1 4719 CTCTATCCAGGGCAGTTGCTACAGTCACTATGTCTTTGGAAACTATTGGAGTACACACTT

BmBRV1-BdEW220 4757 GGAGAGCACCCTCACCTTGAAGGCAGCTACAATGAACAAGCGAAACAGCCAATTGCTCGA

BmBRV1 4758 GGAGAGCACCCTCACCTTGAAGGCAGCTACAATGAACAAGCGAAGCAGCCAATTGCTCGA

SsBRV1 4779 GGAAAGTACACTGACACTTAAGGCTGCTACTATGAACAAGAAGAACAGCCAGCTGCTAGA

BmBRV1-BdEW220 4817 AGAGAAGGAAGAGCGGAGACGGGCTTCATTCAACACAAACACCTACAAGGTTTGTGCTGA

BmBRV1 4818 AGAGAAGGAAGAGCGGAGACGGGCTTCATTCAACACAAACACCTACAAGGTTTGTGCTGA

SsBRV1 4839 GGAGAAAGAAGAGCGAAGAAAATCCTCAATCAATACTGCCACCTACAAAGTGTGTGCAGA

BmBRV1-BdEW220 4877 CTACCCAGACTTCGGTGCGACCCATTCTTGTCGTCAGCAAAGGCTAGTTTTGGAGTGCAT

BmBRV1 4878 CTACCCAGACTTCGGTGCGACCCATTCTTGTCGTCAGCAAAGGCTAGTTTTGGAGTGCAT

SsBRV1 4899 CTACCCAGACTTTGGAGCTACACACTCCTGTCGCCAGCAACGACTGGTTCTCGAGTGCAT

BmBRV1-BdEW220 4937 CCTAGAGGTTGCATGCTCGCAAGGCTTTTTGCCAGACGAAGAGTTTTTGCGCATCCACAA

BmBRV1 4938 CCTAGAGGTTGCATGCTCGCAAGGCTTTTTGCCAGACGAAGAGTTTTTGCGCATCCACAA

SsBRV1 4959 ACTCGAAGTAGCCTGCGCCCAAGGGTTTTTGCCAGATAAGGAATTCTTGCGCATCCACGA

BmBRV1-BdEW220 4997 GTGGTATTCTCAGAGTTTTGAAAACCAGTACTGGATGCGGCCAGATACTTATGAGTGGTA

BmBRV1 4998 GTGGTATTCTCAGAGTTTCGAAAACCAGTACTGGATGCGGCCAGATACTTATGAGTGGTA

SsBRV1 5019 GTGGTATGCTAAGAGTTTTGAGAACCAATACTGGATGAGGCCTGATACCTACGAGTGGTA

BmBRV1-BdEW220 5057 TCGCGCTACTACAGGTATGTTCTCAGGTGTTGTCCAAACTACATTGATTAATACTGTCAT

BmBRV1 5058 TCGCGCTACTACAGGTATGTTCTCAGGTGTTGTCCAAACTACATTGATTAATACTGTCAT

SsBRV1 5079 CAGAGCCACAACAGGAATGTTTTCAGGTGTAGTCCAAACAACTTTGATCAACACCGTCAT

BmBRV1-BdEW220 5117 GAACGGTGCATTGAGGCGGCACTACCTCAAGACTCTCAGCAAGATGGGTAGTCCAGTATC

BmBRV1 5118 GAACGGTGCATTGAGGCGGCACTACCTCAAGACTCTCAGCAAGATGGGTAGTCCAGTATC

SsBRV1 5139 GAATGGCGCACTCCGAAGGCACTACCTTAAGACATTGAGTCTGATGGGCAACCCGGTGTC

BmBRV1-BdEW220 5177 AATGCTCCGCAACTATGAGTTAGGAGACGATGGCTGGGCAGAGTTCCCAACCAGAGAGCA

BmBRV1 5178 AATGCTCCGCAACTATGAGTTAGGAGACGATGGCTGGGCAGAGTTCCCAACCAGAGAGCA

SsBRV1 5199 AATGTTGCGCAACTTTGAGTTGGGAGATGATGGTTGGGCTGAGTTCCCATCGCGAGCGCA

BmBRV1-BdEW220 5237 AGCAGAAAGCTATATTGCTGTCATCCCTCTCTGTGGCAAAGAGCTCAACCCCTTGAAGCA

BmBRV1 5238 AGCAGAAAGCTATATTGCTGTCATTCCTCTCTGTGGCAAAGAGCTCAACCCCTTGAAGCA

SsBRV1 5259 AGCTGAGGCTTACATCGCAGTGATTCCCCTCTGCGGCAAAGAGCTGAATTCGTTGAAGCA

BmBRV1-BdEW220 5297 GTTGATCTCAAGTATATCGAGTGAGTACCTGCGCGAGTGGTATACGAATGGCACAATCTA

BmBRV1 5298 GTTGATCTCAAGTATATCGAGTGAGTACCTGCGCGAGTGGTATACGAATGGCACAATCTA

SsBRV1 5319 GCTGATCTCAAGCATTTCTAGCGAATACTTGCGCGAGTGGTATACTAACGGTACCATCTA

BmBRV1-BdEW220 5357 CGGCTGTGCGTCGCGAGCACTCGCAATGTTGGTAAGTGGAAACGTTGAGAGTAACATTGC

BmBRV1 5358 CGGCTGTGCGTCGCGAGCACTCGCAATGTTGGTAAGCGGAAACGTTGAGAGTAACATTGC

SsBRV1 5379 TGGATGTGCATCGCGTGCACTTGCCATGCTAGTAAGTGGAAATGTAGAGAGCAACATTGC

BmBRV1-BdEW220 5417 GTCCGCAGGAGCTGTACGACTTCGAGAGTTGTACGAAAGTTTCAGCACACTAAGATTGCG

BmBRV1 5418 GTCCGCAGGAGCTGTACGACTTCGAGAGTTGTACGAAAGTTTCAGCACACTAAGATTGCG

SsBRV1 5439 CTCTGCAGGAGCTGTACGCCTACGAGAACTGTACGAAAGCTTCAGCACTTTGAGGCTGCG

BmBRV1-BdEW220 5477 GCACTTCAAACCTCAGATGTGTCAGTACTACTTTGAAGACTTGGCTGTCTACGAAGTGCG

BmBRV1 5478 GCACTTCAAACCTCAGATGTGTCAGTACTACTTTGAAGACTTGGCTGTCTACGAAGTGCG

SsBRV1 5499 TCACTTTAAGCCAGAGATGTGTCAGTATTACTTTGAAGACCTAGCAGTCTACGAAGTAAA

BmBRV1-BdEW220 5537 ACACGGCAAACTTGGCAGAGTGAAGGTGTTACGGTACCTCTATTCAAGCCGAGACCAAAT

BmBRV1 5538 ACACGGCAAACTTGGCAGAGTGAAGGTCTTACGGTACCTCTATTCAAGCCGAGACCAAAT

SsBRV1 5559 ACACGGAAAACTCGGTCGTGTCAAGGTGCTCAGGTACCTTTACTCAAGCCGAGACCAGAT

BmBRV1-BdEW220 5597 GGGTATGGGCCTGTACCCAATCGACCAGATGCCACGAGACCTACAGGACTACGCAAATAT

BmBRV1 5598 GGGTATGGGCCTGTACCCAATCGACCAGATGCCACGAGACCTACAGGACTACGCAAATAT

SsBRV1 5619 GGGCATGGGATTGTATCCTATCCACCAAATGCCTGGAGATTTACAAGACTACGCAAACAT

BmBRV1-BdEW220 5657 GAGCACAGCTGATCAAACTAACCAGACCGGTGATGTTGAGCGCGCTGCAGAGATATTATT

BmBRV1 5658 GAGCACAGCTGATCAAACTAACCAGACCGGTGATGTTGAGCGCGCTGCAGAGATATTATT

SsBRV1 5679 GAACTCAGCGGAGCAGACAAACCAACTGGGTGATGTCGAGCGGGCTGCTGAGATCATCTT

BmBRV1-BdEW220 5717 CGAGCAGAAAGTCTACGGACGCTTTAAGGCATCGAAGGATTACGTTGATGATACTACAAA

BmBRV1 5718 CGAGCAGAAAGTCTACGGACGCTTTAAGGCATCGAAGGATTACGTTGATGATACTACAAA

SsBRV1 5739 TGAGCAAAAGGTCTACGGACGCTTCAAAGCATCGAGGGATTATGTTGATGATACTACCAA

BmBRV1-BdEW220 5777 GCGCTACAACGTTACCTGGCGTCATCAAGGCAAGGCGAGAGCAACAGCCACAATCGCTGC

BmBRV1 5778 GCGCTACAACGTTACCTGGCGTCATCAAGGCAAGGCGAGAGCAACAGCCACAATCGCTGC

SsBRV1 5799 ACGATACAACGTCACCTGGCGCCATCAAGGGAAAGCGAGAGCTACAGCAACTATTGCAGC

BmBRV1-BdEW220 5837 TCAAAATGTAGTTGAAGGGAACAAAACTACCCACGCACAGCATGACGAACTAGAAGTCGC

BmBRV1 5838 TCAAAATGTAGTTGAAGGGAACAAAACTACCCACGCACAGCATGACGAACTAGAAGTCGC

SsBRV1 5859 GCAAAATGTTGTGGAAGGGAACAAAACAACCCAGACGCAGCACAGCGAGCTGGAGGTAGC

BmBRV1-BdEW220 5897 TGTACTGTTGAGTAGTTTCTCGTCAAAAGTTTGGGCTCGTAAGAGTGACATCTTAGTGTG

BmBRV1 5898 TGTACTGTTGAGTAGTTTTTCGTCAAAAGTTTGGGCTCGTAAGAGTGACATCTTAGTGTG

SsBRV1 5919 TGTACTTCTAAGCAGTTTCTCGTCGAAGATCTGGGAGAAGAGAAGCGATGTGCTAGAGAG

BmBRV1-BdEW220 5957 TGCCAAGCCAGCGCGCATCAGTGCGCTGGAAATTGAAAAACAATACTTCAAAGCTACATT

BmBRV1 5958 TGCCAAGCCAGCGCGCATCAGTGCGCTGGAAATTGAAAAACAATACTTCAAAGCTACATT

SsBRV1 5979 AGCAAAGCCTGCAAGTATCAGTGCGAGGGAGATCGAGAAACAGTACTTCAAGGCGACGCT

BmBRV1-BdEW220 6017 TGAAGACCAGCGCTTGCTGTCGCAGATCGGGCAGCTTGCTAAGATCGCAAAATACATGAC

BmBRV1 6018 TGAAGACCAGCGCTTGCTGTCGCAGATCGGGCAGCTTGCTAAGATCGCAAAATACATGAC

SsBRV1 6039 TGAAGATCAGAGGCTCTTGTCTCAGATCGGGCAGTTGGCCAAGATCGCGAAGTATATGAC

BmBRV1-BdEW220 6077 TGAAGAATCGGTATCCAGAATCGCTATGGATATTGCGCTAGAAAATAGCATTCCACTTGA

BmBRV1 6078 TGAAGAATCGGTATCCAGAATCGCTATGGATATTGCGCTAGAAAATAGCATTCCACTTGA

SsBRV1 6099 GGAGGAGTCTATTACTAGGATAGCGATGGATATTGCACTTGAGAACGGTATACCGCTCGA

BmBRV1-BdEW220 6137 AAAAGTGGAGAAAGCGATACGGACATTGAGCTCATTGAAAGGTGAAGGTCTCGATTATCC

BmBRV1 6138 AAAAGTGGAGAAAGCGATACGGACATTGAGCTCACTGAAAGGTGAAGGTCTCGATTATCC

SsBRV1 6159 GAAGGTGCAGAAGGCGATTCGGACTTTGAGTTCGTTGAAGGGCGAGGGGCTTGACTACCC

BmBRV1-BdEW220 6197 TCCAAGGCCACTACTATCACAAGAGTTGATGGGGATATATTCACAGTGGAAAACTGTGGA

BmBRV1 6198 TCCAAGGCCACTACTATCACAAGAGTTGATGGGGATATATTCACAGTGGAAAACTGTGGA

SsBRV1 6219 CCCAAGACCGTTACTCTCACAGGAGTTGATGGGGATATACTCACAATGGAGTGCAGTCGA

BmBRV1-BdEW220 6257 CAAGAGGGAAGATGATATGTATTTGCCGGAGTGGTTGATGGTGCTAGCACCACATTACCG

BmBRV1 6258 CAAGAGGGAAGATGATATGTATTTGCCGGAGTGGTTGATGGTGCTAGCACCACATTACCG

SsBRV1 6279 TAAGCGAGAGAGTGGCGCGTATCTACCAGATTGGTTGATTACGTTAGCACCACACTACCG

BmBRV1-BdEW220 6317 CACATAAACGTTAACACACGCAGTGTTGAGGGAAGGGATTGAGAATT-AAGCAGAAACTA

BmBRV1 6318 CACATAAACGTTAACACACGCAGTGTTGAGGGAAGGGATTGAGAATT-AAGCAGAAACTA

SsBRV1 6339 CACATAAACGTTAACACATGCAGTGTTGAGGAGAGGGATTGAGAATTAAAGCAGAAACTA

BmBRV1-BdEW220 6376 TGTTTCATACGAGTTAAGGGTTAGTCCCAAAAATAGATCCCAGAGGATCTACAAATAGT

BmBRV1 6377 TGTTTCATACGAGTTAAGGGTTAGTCCCAAAAATAGATCCCAGAGGATCTACAAATAGC

SsBRV1 6399 AGTTTCATACGAGTTAAGGGTTAGTCCCAAAAATAGATCCCAGAGGATCTACAAATAGC
