# Supplementary material for: Characterization of a Botybirnavirus Conferring Hypovirulence in the Phytopathogenic Fungus Botryosphaeria dothidea
Source: Viruses. 2019 Mar 17;11(3):266. doi: 10.3390/v11030266 (PMC6466033; doi:10.3390/v11030266)
Supplement: Supplementary file 1 [file viruses-11-00266-s001.zip › viruses-449497-supplementary/Manuscript supplementary Figure 2.docx]

**Supplementary**

**Figure S2.** Multiple-sequence alignments of the sequences of P1 encoded by BmBRV1-BdEW220, BmBRV1 and SsBRV1. Abbreviations: BmBRV1-BdEW220, Bipolaris maydis botybirnavirus 1 strain BdEW220; BmBRV1, Bipolaris maydis botybirnavirus 1; SsBRV1, Sclerotinia sclerotiorum botybirnavirus.

BmBRV1-BdEW220 1 MS--LNFASISDFASAASIALPLEMWIEIVANLSTWDAACIVKNIPVCRAPVYTALLARA

BmBRV1 1 MS--LNFASISDFASAASIALPLEMWIEIVANLSTWDAACIVKNIPVCRAPVYTALLARA

SsBRV1 1 MSFSNNIVSVSALPTLAT--LPYEMWSSVVSNLSTWDAACIVKNIPMCQSLLYQALLARA

BmBRV1-BdEW220 59 NQEFNFGCCVEEIISSIPCSHSRPCEIVSKDAQFSLACVEHISASCGSSCEKVLISEYDL

BmBRV1 59 NQGFNFGCCVEEIISSIPCSHSRPCEIVSKDAQFSLACVEHISASCGSSCEKVLISGYDL

SsBRV1 59 NLDYNFGCTHEKIISTMPCNHPRTCEVLPKGGHFAFVCAEHIVGTCSASCDSVLMSADDV

BmBRV1-BdEW220 119 GVRLKHREAERAQRRLDRIYGPLATEHDMMVIDDQHSETSSYRDYLRYSAEIAYTEEDLH

BmBRV1 119 GVRRKHREAERAQRRLDRIYGPLATEHDMMVIDDQHSETSSYRDYLRYGAEIAYTEEDLH

SsBRV1 119 YSRFKRYEESRLQRKRDHMYGPFALEHDMMVIDDQNAATSSHRDYMRYRAEMVYVEEDLA

BmBRV1-BdEW220 179 ISSLFEAVSFQPEGSDVEKTRGGMDAADAGPSESAQAAPTPVDDEVPQDGPVTGEPTVGL

BmBRV1 179 ISSLFEAVSFQPEGSDVEKTRGGMDAADAGPSESAQAAPTPVDDEVPQDGPVTGEPTVGL

SsBRV1 179 LGTLFGGDLASTDTLVVERTRGGMDAAGSGAPEASAAAPTPVEDEVPQDGPVNEEPVVGL

BmBRV1-BdEW220 239 PPGSEEPGHPGPDVPLDPRVTNGDDFGHIPNTEDASDXVDAVATNGWFEYPYATGGGQAT

BmBRV1 239 PPGSGEPGHPGPDVPLDPRVTNGDDFGHIPNTEDASDPVDAVATNGWFEYPYATGGGQTT

SsBRV1 239 PPGSEEPSHPGPDVPLDPRVTNGDDFGNIPNTEDASDPVDAVATNGWFEYPYAEGSGQVA

BmBRV1-BdEW220 299 SYAGLITQADPTSEYVTNISDRFRSISPQFEELERNIRVTSGFGVQSYLVQTLWGFGPRG

BmBRV1 299 SYAGLITQADPTSEYVTNISDRFRSISPQFEELERNIRVTSGFGVQSYLVQTLWGFGPRG

SsBRV1 299 SYAGLITQANPSSEYVTNISDRFRNISPQFEELERNIRVTSGSGVQSYLVQTIWGFGPRG

BmBRV1-BdEW220 359 ASSLVTQSANADSRTVAFWTTNPKSEIIPVSEDTIMYDTLAGLSIEGQMVRLNTTFNGNM

BmBRV1 359 ASSLVTQSANADSRTVAFWTTNPKSEIIPVSEDTIMYDTLAGLSIEGQMVRLNTTFNGNM

SsBRV1 359 AASLMTQSANADTRTVAFWTTNPQFEIVPVSEDTIMYDTLAGLSVEGQMVRLNTTFNDNM

BmBRV1-BdEW220 419 VTDLYNSIGDRAIAERYYDHVVTAAVAGSNYVAFLTMCYTRLVSLKIMAEQNQPATMRVE

BmBRV1 419 VTDLYNSIGDRAIAERYYDHVVTAAVAGSNYVAFLTMCYTRLVSLKIMAEQNQPATMRVE

SsBRV1 419 ITDLYNSVGDRAIAQRYYDHVVTAAVAGSNYVAFLTICYTRLVALKIMSEQNQPAQMRVS

BmBRV1-BdEW220 479 GDTLNANILLDNVVANSVTRRVADAVLRSKPTNAVMLPHGSNDLDVETMLYLMGHGRVXK

BmBRV1 479 GDTLNANILLDNVAANSVTRRVADAVLRSKPTNAVMLPHGSNDLDVETMLYLMGHGRVIK

SsBRV1 479 GDTLNANILLDTVAANPVTRRVADAVLRSKPTNAVMLPHGSNDLDVETMLYLMGHGRVIK

BmBRV1-BdEW220 539 GVATEDEEIAVFSPFDRFHTDSNFKLVGLVGENAIGHFPAGNLEFQIDFGQAFDLLNRYV

BmBRV1 539 GVATEDEEIAVFSPFDRFHTDSNFKLVGLVGENAIGHFPAGNLEFQIDFGQAFVLLNRYV

SsBRV1 539 SVATDEDEVAVFSPFDRFHTDSNFKLLGVVGERAIGNFPPGNLEFQIDFGQAFDLLNRYI

BmBRV1-BdEW220 599 NQNDLWDQFAIARNIALGMIFSRSFSASVGLPKPYHSRDLALNRTSTGANQHGRRRVMEF

BmBRV1 599 NQNDLWDQFAIARNIALGMIFSRSFSASVGLPKPYHSRDLALNRTSTGANQHGRRRVMEF

SsBRV1 599 NQNDLWDQFAIARNIALGMIFSRTFSSSVGLPKPYHSRDLALNRTHTGANQHGRRRVMEF

BmBRV1-BdEW220 659 KESFHALVXSGTWHCAAMEETLFESVVNVMEETAGIGPRAPNFYATIDTMQDDFDLEYKT

BmBRV1 659 KESFHALVVSGTWHCAAMEETLFESVVNVMEETAGIGPRAPNFYATIDTMQDDFDLEYKT

SsBRV1 659 KESFHAVVASGTWHCAAMEETLFESVVNVMEETAGIGPRAPNFYATIDTMQDDFDLEYKT

BmBRV1-BdEW220 719 AMVCLPVVERMTGTPSNHIHQYVSKSNTAFMKAISFGWESXPIRISSYLALEITPEDKNF

BmBRV1 719 AMVCLPVVERMTGTPSNHIHQYVSKSNTAFMKAISFGWESKPIRISSYLALEITPEDKNF

SsBRV1 719 AMVCLPVVERMTGTSSSHIHQYVSKSNVAFMKAISFGWESKPIRISSYLALEITPEDKNF

BmBRV1-BdEW220 779 KFLFDKSARTALLKQEKYTWRESVLTSFVCHSQWGPSEQHRSYGEFYDDGISALREQTPF

BmBRV1 779 KFLFDKSARTALLKQEKYTWRESVLTSFVCHSQWGPSEQHRFYGEFYDDGISALREQTPF

SsBRV1 779 KFLFDKSARTALLKQEKFTWREAVLTSFVCHSQWGPSEQHRFYGEFYDDNISDLREQVPF

BmBRV1-BdEW220 839 YGYLGRSQLEHLEGTPTIWNTSTAATRIEFTKPASLARATHGLNEDEASQVDSLWATMKA

BmBRV1 839 YGYLGRSQLEHLEGTPTIWNTSTAATRIEFTKPASLARATHGLNEDEASQVDSLWATMKA

SsBRV1 839 YGYLGRSQLEHLEGSPTIWNTSTAATPLEFTKPKSLTRVTRDLNEEEAMQVDSLWATMKA

BmBRV1-BdEW220 899 ALEAASADNDAEESDDDEFVSPPQPTANPTQKGRRFEVREPKGKEIARVVPEPEHQRAGT

BmBRV1 899 ALEAASADNDAEESDDDESVSPPQPTANPTQKGRRFEVREPKGKEIARVVPEPEHQRAGT

SsBRV1 899 ALEAASAENDAEEDEDDEEFEYPQPTANPTGQGQRFELHEPKGKEVERSIPEPEQQRAGT

BmBRV1-BdEW220 959 DRPIPTSQWQKPKHTAKAVTAANQNGLVQKNYFQKLTPAPTLIGLNPNGGTPGAQAQGPS

BmBRV1 959 DRPIPTSQWQKPKHTAKAVIAANQNGLVQKNYFQKLTPAPTLIGLNPNGGTPGAQAQGPS

SsBRV1 959 DRPIPASQWQRPKNAARVSASVEKTQVQARNYFQGLATPSRLIGFNQSTPTPEMVPQQRN

BmBRV1-BdEW220 1019 RQLTTSTIKRMTPKQSDRADHAYLDSQIKRVTSERRLFEQANRGGATRSGXKKKEKVIPA

BmBRV1 1019 RQLTTSTIKRMTPKQSDRADHAYLDSQIKRVTSERRLFEQANRGGATRSGSKKKEKVIPA

SsBRV1 1019 SPLAIKTLKKVSDRQKDYEEHVYLDTQVKKTAAERRQFEQYHRGGMQKKANRKDKRVAPA

BmBRV1-BdEW220 1079 VLRDEVEQLCSRMVTDLRFRVDLLTRLPSTEDESKAVDFLYPRGKDGSLKRAVYTIGTLL

BmBRV1 1079 VLRDEVEQLCSRMVTDLRFRVDLLTRLPSTEDESKAVDFLYPRGKDGSLKRAVYTIGTLL

SsBRV1 1079 QVRDEVEQLCSRMVTDLRFRTELLTRLPATENESKAVDFLYPRGKDGSLKRAVYTIGTLL

BmBRV1-BdEW220 1139 RKLKTDRKLTVEQQADISLFLNSNVGGKNAWAVAIVMFITLNTLTPECYQMLKSYGLLTT

BmBRV1 1139 RKLKTDRKLTVEQQADISLFLNSNVGGKNAWAVAIVMFITLNTLTPECYQMLKSYGLLTT

SsBRV1 1139 RKLRCDEKLTATQKSDINLFLTTNVGGKNAWAVAVVMFISLNTLTPDCYEMLKSYGFLTT

BmBRV1-BdEW220 1199 QYNHWNDKWSRINDMFRNQMDSETWGFSETDFPQCLYIAGFVGRPHREADWEAENIKRSA

BmBRV1 1199 QYNHWNDKWSRINDMFRNQMDSETWGFSETDFPQCLYIAGFVGRPHREADWEAENIKRSA

SsBRV1 1199 PYNQWNDKWSKINDLFRNQMDSETWLYSETDFPQCLYIAGFVGRPHREADWEAENAKRSA

BmBRV1-BdEW220 1259 EPKPIKKYTKSGFQDMSEEDERVMILDFLYSEASFRIKRVQGFERWYRNRAEWMIKGSMS

BmBRV1 1259 EPKPIKKYTKSGFQDMPEEDERVMILDFLYSEASFRIKRVQGFERWYRNRAEWMIKGSMS

SsBRV1 1259 EPKPIRKFTKSGFVDMSEEDEKRMILDFLYSEATFRIKRVQGFERWYRSRAEWMIKGSMS

BmBRV1-BdEW220 1319 GEKTILDTEPVVMAKLKDLGLKVDGHANKMHIAEKVDYTWMIAVLDMDPVHLAKMHTKGQ

BmBRV1 1319 GEKTILDTEPVVMAKLKDLGLKVDGHANKMHIAEKVDYTWMIAVLDMDPVHLAKMHTKGQ

SsBRV1 1319 GEKTILDTEPVVMAKLKDLGLKVDGHANKMHIAEKVDYTWMIAVLDMDPVHLAKMHTKGQ

BmBRV1-BdEW220 1379 ENGKVRSIQGSCYSHYVFGNYWSTHLESTLTLKAATMNKRNSQLLEEKEERRRASFNTNT

BmBRV1 1379 ENGKVRSIQGSCYSHYVFGNYWSTHLESTLTLKAATMNKRSSQLLEEKEERRRASFNTNT

SsBRV1 1379 ENGKIRSIQGSCYSHYVFGNYWSTHLESTLTLKAATMNKKNSQLLEEKEERRKSSINTAT

BmBRV1-BdEW220 1439 YKVCADYPDFGATHSCRQQRLVLECILEVACSQGFLPDEEFLRIHKWYSQSFENQYWMRP

BmBRV1 1439 YKVCADYPDFGATHSCRQQRLVLECILEVACSQGFLPDEEFLRIHKWYSQSFENQYWMRP

SsBRV1 1439 YKVCADYPDFGATHSCRQQRLVLECILEVACAQGFLPDKEFLRIHEWYAKSFENQYWMRP

BmBRV1-BdEW220 1499 DTYEWYRATTGMFSGVVQTTLINTVMNGALRRHYLKTLSKMGSPVSMLRNYELGDDGWAE

BmBRV1 1499 DTYEWYRATTGMFSGVVQTTLINTVMNGALRRHYLKTLSKMGSPVSMLRNYELGDDGWAE

SsBRV1 1499 DTYEWYRATTGMFSGVVQTTLINTVMNGALRRHYLKTLSLMGNPVSMLRNFELGDDGWAE

BmBRV1-BdEW220 1559 FPTREQAESYIAVIPLCGKELNPLKQLISSISSEYLREWYTNGTIYGCASRALAMLVSGN

BmBRV1 1559 FPTREQAESYIAVIPLCGKELNPLKQLISSISSEYLREWYTNGTIYGCASRALAMLVSGN

SsBRV1 1559 FPSRAQAEAYIAVIPLCGKELNSLKQLISSISSEYLREWYTNGTIYGCASRALAMLVSGN

BmBRV1-BdEW220 1619 VESNIASAGAVRLRELYESFSTLRLRHFKPQMCQYYFEDLAVYEVRHGKLGRVKVLRYLY

BmBRV1 1619 VESNIASAGAVRLRELYESFSTLRLRHFKPQMCQYYFEDLAVYEVRHGKLGRVKVLRYLY

SsBRV1 1619 VESNIASAGAVRLRELYESFSTLRLRHFKPEMCQYYFEDLAVYEVKHGKLGRVKVLRYLY

BmBRV1-BdEW220 1679 SSRDQMGMGLYPIDQMPRDLQDYANMSTADQTNQTGDVERAAEILFEQKVYGRFKASKDY

BmBRV1 1679 SSRDQMGMGLYPIDQMPRDLQDYANMSTADQTNQTGDVERAAEILFEQKVYGRFKASKDY

SsBRV1 1679 SSRDQMGMGLYPIHQMPGDLQDYANMNSAEQTNQLGDVERAAEIIFEQKVYGRFKASRDY

BmBRV1-BdEW220 1739 VDDTTKRYNVTWRHQXKARATATIAAQNVVEGNKTTHAQHDELEVAVLLSSFSSKVWARK

BmBRV1 1739 VDDTTKRYNVTWRHQGKARATATIAAQNVVEGNKTTHAQHDELEVAVLLSSFSSKVWARK

SsBRV1 1739 VDDTTKRYNVTWRHQGKARATATIAAQNVVEGNKTTQTQHSELEVAVLLSSFSSKIWEKR

BmBRV1-BdEW220 1799 SDILVCAKPARISALEIEKQYFKATFEDQRLLSQIGQLAKIAKYMTEESVSRIAMDIALE

BmBRV1 1799 SDILVCAKPARISALEIEKQYFKATFEDQRLLSQIGQLAKIAKYMTEESVSRIAMDIALE

SsBRV1 1799 SDVLERAKPASISAREIEKQYFKATLEDQRLLSQIGQLAKIAKYMTEESITRIAMDIALE

BmBRV1-BdEW220 1859 NSIPLEKVEKAIRTLSSLKGEGLDYPPRPLLSQELMGIYSQWKTVDKREDDMYLPEWLMV

BmBRV1 1859 NSIPLEKVEKAIRTLSSLKGEGLDYPPRPLLSQELMGIYSQWKTVDKREDDMYLPEWLMV

SsBRV1 1859 NGIPLEKVQKAIRTLSSLKGEGLDYPPRPLLSQELMGIYSQWSAVDKRESGAYLPDWLIT

BmBRV1-BdEW220 1919 LAPHYRT

BmBRV1 1919 LAPHYRT

SsBRV1 1919 LAPHYRT
