# Supplementary material for: Characterization of a Botybirnavirus Conferring Hypovirulence in the Phytopathogenic Fungus Botryosphaeria dothidea
Source: Viruses. 2019 Mar 17;11(3):266. doi: 10.3390/v11030266 (PMC6466033; doi:10.3390/v11030266)
Supplement: Supplementary file 1 [file viruses-11-00266-s001.zip › viruses-449497-supplementary/Manuscript supplementary Figure 3.docx]

**Supplementary**

**Figure S3.** Multiple-sequence alignments of the dsRNA2 segment of BmBRV1-BdEW220, BmBRV1 and SsBRV1. Abbreviations: BmBRV1-BdEW220, Bipolaris maydis botybirnavirus 1 strain BdEW220; BmBRV1, Bipolaris maydis botybirnavirus 1; SsBRV1, Sclerotinia sclerotiorum botybirnavirus.

BmBRV1-BdEW220 1 GCAATAAAAGGCGAAGCCGGGAGGCTTTTATTTTATTGCCCAGTTT-TTCATCTGGTTAA

BmBRV1 1 GCAATAAAAGGCGAAGCCGGGAGGCTTTTATTTTATTGCCCAGTTTCTTCATCTGGTTAA

SsBRV1 1 GCAATAAAAAGCACAGCCGGAAGGCTTTCTTTTTATTGCCCAGTTT-TTGATCTGGTCAA

BmBRV1-BdEW220 60 AATGAAGTCGGCTATC--CAGCCAGCGGAGAGTGGAACTGGTCCTCTTCGTACGAAACCA

BmBRV1 61 AATGAAGTCGGCTATC--TAGCCAGCGGAGAGTGGAACTGGTCCTCTTCGTACGAAACCA

SsBRV1 60 AATCAAACTGGCTTGCCATAGCCACCAGAAGATGGAACTGGTCTCTTCTGTTCTACACCA

BmBRV1-BdEW220 118 AACCGAGCTCCCACCCGGTGAGGTAATCCAGTGGCAATGCGTGGGGGAGACCTCGGCCT-

BmBRV1 119 AACCGAGCTCCCACCCGGTGAGGTAATCCAGTGGCAATGCGTGGGGGAGACCTCGGCCT-

SsBRV1 120 ATCCGAGCTCCCACCCGGTGCGGTAATCCAGTGGTAATGCGTGGGGGAGACCTCGGTTTT

BmBRV1-BdEW220 177 ----------CGGCCGAGTGATTGTGCAGAACGGGTGGTGATGTCAGCAATGCTCACCGT

BmBRV1 178 ----------CGGCCGAGTGATTGTGCAGAACGGGTGGTGATGTCAGCAATGCTCACCGT

SsBRV1 180 AAGCTTAAAGCAGCCGAGTGATTGTGCAGAACGGGTGGTGATGTCAGCAATGCTCACCGT

BmBRV1-BdEW220 227 ACATGTCCGTGCTAGGGAGAATGTCCTGTATCCAGCAGGAAGATATCCCGCGTGTAACGA

BmBRV1 228 ACATGTCCGTGCTAGGGAGAATGTCCTGTATCCAGCAGGAAGATATCCCGCGTGTAACGA

SsBRV1 240 ACATGTCCGTGCTAGGGGGAATGTCCTGTAACCAGCAGGAAGATACCCCGCGTGTAACGA

BmBRV1-BdEW220 287 CTGCAAGCACTTTGTCTCCTACGTAGAACAGTCTATCACCTAGCGGCGCTGATCGGCCGA

BmBRV1 288 CTGCAAGCACTTTGTCTCCTACGTAGAACAGTCTATCACCTAGCGGCGCTGATCGGCCGA

SsBRV1 300 CTGCAAGCACTTTGTCTCCTACGTAGAACAGTCTACCACCTAGCGGCGCTGATCGGCCGA

BmBRV1-BdEW220 347 TACAGTAGGTGGTGTGTAGGCTTGCC-AGCCCGACCCCCGCCACAGACATAGCTTCCAAA

BmBRV1 348 TACAGTAGGTGGTGTGTAGGCTTGCC-AGCCCGAACCCCGCCACAGACATAGCTTCCAAA

SsBRV1 360 TACAGTAGGTGGTGTGTAGGCTTACCAAACCCGACCCACGCCACAG-CAAAGTTACCAAG

BmBRV1-BdEW220 406 CAACTACACACACACAATACAGCAGAGTGAGCTAATCCAGCAGCTGTATT-ACCACATTA

BmBRV1 407 CGACTACACACACACAACACAGCAGAGTGAGCTAATCCAGCAGCTGTATT-ACCACATTA

SsBRV1 419 C--CAACACACATACAATATAGTAGAGTGAGCTAATCCAGCAACTATATTGAACAATTTA

BmBRV1-BdEW220 465 CAGTAGATCAACTTTTGAATACTGTTCACGATCAAACAAACTACTTACTTCATCAAACCG

BmBRV1 466 CAGTAGATCAACTTTTGAATACTGTTCACGATCAAACAAACTACTTACTTCATCAAACCG

SsBRV1 477 CAGTAGATCAACTTTTGAATACTGTTTTCGATCACATACACAAAATAACATATAAAACAC

BmBRV1-BdEW220 525 CAAATCTTTCTAAATAAAACATTTAAAACCAAAAACAATTCA-AATGTCTTACCGTTCCA

BmBRV1 526 CGAATCTTTATAAATAAAACATTTAAAACCAAAAACAATTCA-AATGTCTTATCGTTCCA

SsBRV1 537 AAGAAC----AAACTAAAACAAATAAAACCAAAAACAATTCATCATGTCTTACCGATCCA

BmBRV1-BdEW220 584 ATTCTGTCTCTTTGCATTCTGTACCCTCCAAGCAGTTGCTTGGCTGTACCTCGATGTTGT

BmBRV1 585 ATTCTGTCTCTTTGCATTCTGTACACTCCAAGCAATTGCTTGGCTGTACCTCGATGTTGT

SsBRV1 593 ACTCTATTTCTTTGAATTCTGTCCGCTCCAAGCATATGCGCGGAGTGACTTTTGTTTTAC

BmBRV1-BdEW220 644 TTGTTGAGAAGACCCGTGGTGGCGTCAATCTTAAGAAGAAGATGCCCACACGTGTTGAAG

BmBRV1 645 TTGTTGAGAAGACCCGTGGTGGCGTCAATCTTAAGAAGAAGATGCCCACACGTGTTGAGG

SsBRV1 653 CTGTGGAGAAGACTCGCGGGGGTATCAGTCTTAAAAAGAAGATGCCTACACGTGTTGAAG

BmBRV1-BdEW220 704 ACGCTGGTATCCGTGCTTCCGGGAAACTCGGAGGTAAGGAGACCAGCGCAACTGCTTCAG

BmBRV1 705 ACGCTGGTATCCGTGCTTCCGGAAAACTCGGAGGTAAGGAGACCAGCGCAACTGCTTCAG

SsBRV1 713 ACGCTGGTATCCGTGCTTCCGAACAACTCGGAGGCAAGGAGACCAGCGCTACTGCTTCAG

BmBRV1-BdEW220 764 AAGCCAGCCCTCTGGGCGGCCTCTTCGATGCAGTGAGCGCCCTTTCGATGGAATCTGCTG

BmBRV1 765 AAGCCAGCCCTCTGGGCGGCCTCTTCGATGCAGTGAGCGCCCTTTCGATGGAATCTGCTG

SsBRV1 773 AAGCTAGCCCTCTGGGCGGCCTCTTTGACGCAGTAACTTCTCTCGCAACGGAGGTTGCCG

BmBRV1-BdEW220 824 ATCCTCAGTTCGGTAACCCATCGAATGTTCTGGAAGGTGAGAGTTATTCTGAGTTGTGTC

BmBRV1 825 ATCCTCAGTTCGGTAACCCATCGAATGTTCTGGAAGGTGAGAGTTATTCTGAGTTGTGTC

SsBRV1 833 ACCCTCAATTCGGCACTCCGTCAACCATCTTGGAAGGTGAGAGTTACTCCGAACTGTGCT

BmBRV1-BdEW220 884 TTACACAATCCATACTCTCGCGTATGCCAGACATCAACTTCAAGAACCCCTTCTTGGTTC

BmBRV1 885 TTACACAATCCATACTCTCGCGTATGCCAGACATCAACTTCAAGAACCCCTTCTTGGTTC

SsBRV1 893 TGACACAGTCGATTCTCTCTCGCATGCCAGATATCAACTTCAAGAATCCCTTTTTGGTCC

BmBRV1-BdEW220 944 AAGCACCAAGTGCTAAAGACCAGGTAGTCTACATGGCATGGGGCTACAAGTTGAAGAGTT

BmBRV1 945 AAGCACCAAGTGCTAAAGACCAGGTAGTCTACATGGCCTGGGGCTACAAGTTGAAGAGTT

SsBRV1 953 AGGCTCCGACTGCCAAATACCAAACTGTCTACATGGCATGGGGTTACAAGTTGAAGAGCT

BmBRV1-BdEW220 1004 TGCCCAGTTCGCAGAAAGGCTCAGCTTACGCTGACAAGTATACAGTGAAATTGTGGGCGA

BmBRV1 1005 TGCCCAGTTCGCAGAAAGGCTCAGCTTACGCTGACAAGTATGCAGTAAAATTGTGGGCGA

SsBRV1 1013 TGCCGAGCTCACAGAAAGGCTCTGCCTATGCTGACAAATACGGCGTAAAGTTGTGGGCTA

BmBRV1-BdEW220 1064 AGACCAACGAGGTGCGAGTGCAGTACCCTGTGATTGCTACATCTGTTGGTTACCGCAATA

BmBRV1 1065 AGACCAACGAAGTGCGAGTGCAGTACCCTGTGATTGCTACATCTGTTGGCTACCGCAATA

SsBRV1 1073 AGACGAAGGAGGTCCGGGTGAACTACCCTGTGATCGCCACTTCAACCGGGTACCGCAACC

BmBRV1-BdEW220 1124 AGATAACCACAGAGTATGAGCAACGAGCGACCGGCAACCTTGTCACAACACAACGTGTGG

BmBRV1 1125 AGATAACCACAGAGTATGAGCAACGAGCGACCGGCAACCTTGTCACAACACAACGTGTGG

SsBRV1 1133 GCATTACAACTGAGTACGAACAACGTGCTACCGGTAACCTTGTCACAACCCAGCGTGTGG

BmBRV1-BdEW220 1184 ACTCTCGCGAGTTCCAGGCCAGTTTTGATGAGATGAAAGGCTTCCCAATTCAGTTCAGGC

BmBRV1 1185 ACTCTCGCGAGTTCCAGGCCAGTTTTGATGAGATGAAAGGCTTCCCAATTCAGTTCAGGC

SsBRV1 1193 ATTCTCGCGAGTTCCAAGCTAGGTTTGATGAGATGAAAGGCTTCCCGATTGAATTCCGTC

BmBRV1-BdEW220 1244 TCGGTGGTAGCGCTAAAAGCGTTGATGGCGACAACCACATTGCGTGGTTGGTCATCGCCA

BmBRV1 1245 TCGGTGGTAGCGCGAAAAGCGTTGATGGCGACAACCACATTGCGTGGTTGGTCATCGCCA

SsBRV1 1253 TCGGTGGTACTGCAAAGAGTGTAGACGGTGATAACCACGTAGCGTGGCTCACCATCGCTA

BmBRV1-BdEW220 1304 TCCTCAGGCTGTTTGCGCTTAAGCAAGCACAAGAGACCAATTCCCGAGGGCACATCACGA

BmBRV1 1305 TCCTCAGGCTGTTTGCGCTTAAGCAAGCACAAGAGACCAATTCCCGAGGGCACATCACGA

SsBRV1 1313 TTCTTAGACTCCTAGCTCTCAAACAAGCCCAGGAGACTAACTCCCGAGGCCACATTGAAA

BmBRV1-BdEW220 1364 TGTCTACTGCTATTGCTAGCTCATTTAGCATCAATCTCGAGGACATGGTACAGGCGCGGT

BmBRV1 1365 TGTCTACTGCTATTGCTAGCTCACTTAGCATCAATCTCGAGGACATGGTACAGGCGCGGT

SsBRV1 1373 TGACCTCCGGTATTGCCAACCAGTTTGGCATCAACCTTGAAGACATGGTGCAAGCACGGT

BmBRV1-BdEW220 1424 CTTCTGCCAGCCACAGCGTAGCCACAGCTTTGGCTAACGCCTGGTTTGGCGCCGCTAAAC

BmBRV1 1425 CTTCTGCCAGCCACAGCGTAGCCACAGCTTTGGCTAACGCCTGGTTTGGCGCCGCTAAAC

SsBRV1 1433 CTTCCTCTAGCCACAGCGTGGCACCTGCCTTGGCTAACGCTTGGTTTGGCTCTGCCAAGT

BmBRV1-BdEW220 1484 CTGGTGGCAGGCCTGGTGCTGTGGAAGCCGTCCCAGACAAAATGTTTGAGATGGTTTTGC

BmBRV1 1485 CTGGTGGCAGGCCTGGTGCTGTGGAAGCCGTCCCAGACAAAATGTTTGAGATGGTGTTGC

SsBRV1 1493 CAGGCGGTAAGCCAGGAGCTGTAGAGGCCGTGCCAGACCGTGTCTTTGAGATGGTATTGC

BmBRV1-BdEW220 1544 CTAGCACTGCTGCAGACGTGAGCGAAGTAGTGTATTTGGCATACCTTTCTGGTATGCTAG

BmBRV1 1545 CTAGCACTGCTGCAGATGTAAGCGAAGTAGTGTATTTGGCATACCTTTCTGGTATGCTAG

SsBRV1 1553 CCAGCAATGCCGCAGACGCCAGCGAAGTAGTTTATCTAGCTTACCTCTCTGGTATGCTTG

BmBRV1-BdEW220 1604 ATAACTCTATGCGATGGAGGAACGGCGATGAGGAACTTGGGATCCAGCCGATGTTCTCAT

BmBRV1 1605 ATGACTCTATGCGATGGAGGAACGGCGATGAGGAACTTGGGATCCAGCCGATGTTCTCGT

SsBRV1 1613 ACGACTCCATGCGCTGGAGGAACGGAGATGAAGAGTTAGGTATCCAACCTATGTACTCAG

BmBRV1-BdEW220 1664 CGATCCGCTCCACCGCAACAGAGCGGATGAAAATCCCTCTTGTTAACGCATCTCGTGCGA

BmBRV1 1665 CGATCCGCTCCTCCGCAACAGAGCGGATGAAAATTCCTCTTGTTAACGCATCTCGTGCGA

SsBRV1 1673 CAATCCGCTCCTCAGCAACCGAGCGTATGAAGATCCCTTTCGTCAATGCGTCCCGTGCTA

BmBRV1-BdEW220 1724 TCCCGATGGCAGATTTGCGAGGCTATGACTCTGATCTAGAGTTAGGCCGTGCTGAAGCTG

BmBRV1 1725 TCCCAATGGCAGATTTGCGAGGCTATGATTCTGATCTAGAGTTAGGCCGTGCTGAAGCTG

SsBRV1 1733 TTTCAATGAATGACTTGAAAAAATTCAACGCCGACATTGAACTTGGTCGGGCTGAAGCAG

BmBRV1-BdEW220 1784 TCTTCAACTCGTATGTGCGTCGGCACGAACTGCAGAGCCAAGTTAATGTTGCTCGCAGGA

BmBRV1 1785 TCTTCAACTCGTATGTGCGTCGACACGAACTGCAGAGCCAAGTGAATGTTGCTCGCAGGG

SsBRV1 1793 TTTTTAACTCGTACGTTAGGCGCCACGAGTTGCAAAGCCAGGTGAATGTCGCTCGCCGGA

BmBRV1-BdEW220 1844 TAGCACTATTGGCGATGCTCGATACCCACAGCAGCAAGAGTTCTCGTATGCTTCTCGGCT

BmBRV1 1845 TAGCACTATTGGCGATGCTCGATACCCACAGCAGCAAGAGTTCTCGTATGCTTCTCGGCT

SsBRV1 1853 TTGCCCTCTTGGCCATGCTCGATACCCATAGTAATAAGGGCTCGCGCATGCTTCTTGGCT

BmBRV1-BdEW220 1904 TACCTAAGCCTTGCCATGTCGTCGAGTACGATCTTTGGGTCGACCCCAAGCATTTCAGCA

BmBRV1 1905 TACCTAAGCCTTGCCATGTCGTCGAGTACGATCTTTGGGTCGACCCCAAGCATTTCAGCA

SsBRV1 1913 TACCTAAGCCCTGCCATGTGCTCGAGTATGACTTGTGGGTCGACCCGAAACACTACAGCA

BmBRV1-BdEW220 1964 CTTCCCCGCTGAGTCTCATCGCTACAAGCGAGAGTGCGGGTTTGCTTATGGCTTTGAGCC

BmBRV1 1965 CCTCCCCGCTGAGTCTCATCGCTACAAGCGAGAGTGCGGGTTTGCTTATGGCTTTGAGCC

SsBRV1 1973 CATCACCCCTGAACCTCATTGCGACTAGCGAGAGTGCGGGTTTGCTTATGGCATTAAGCC

BmBRV1-BdEW220 2024 AAATGCAAGCTACAATGCGGAATGATATATTGGTCATGAAATTGATTGACCACCTCGAGA

BmBRV1 2025 AAATGCAAGCTACAATGCGGAACGATATATTGGTCATGAAATTGATTGACCACCTCGAGA

SsBRV1 2033 AAATGCAAGCCACAATGCGGAATGACATCCTTGTCATGAAGCTCATTGACCATCTCGAGA

BmBRV1-BdEW220 2084 GTAAGGGCACACCAGTGCTTTCGTCTCTCGCCTACGAATCGCTGAGGGATTGGATCTATG

BmBRV1 2085 GTAAGGGCACACCAGTGCTTTCGTCTCTCGCCTACGAATCGCTGAGGGATTGGATCTATG

SsBRV1 2093 GCCGTGGTACCCCTGTCCTCTCCTCTCTTGCCTACGAGTCTCTTCGCGACTGGATCTACG

BmBRV1-BdEW220 2144 ATGCAATGCCCAGTGGATTCTCCTCTTGGTCAAGAGGGTGGTTGAGTACCGTGACAGGCA

BmBRV1 2145 ATGCAATGCCCAGTGGATTCTCCTCTTGGTCAAGAGGGTGGTTGAGTACCGTGACAGGCA

SsBRV1 2153 ATGCCATGCCGAGCGGCTTTTCATCCTGGTCTAGAGGATGGTTGAACACCGTAACTGGTG

BmBRV1-BdEW220 2204 CAAGCCCACCTGAAATCAGAAGGTTGGCAAACGATAGCGTGCATGGATTCTGGACTGGCT

BmBRV1 2205 CAAGCCCACCTGAAATCAGAAGGTTGGCAAACGATAGCGTGCATGGATTCTGGACTGGCT

SsBRV1 2213 GTAGCCCTCCTGAGATCAGGCGACTCGCCAACGACAGCGTCCATGGCTTCTGGACCGGCC

BmBRV1-BdEW220 2264 TGAATGCCACGCTCTTATCCGGCACAGTTCATGTGAGTGCAATGCTCTATTACGGGGAGA

BmBRV1 2265 TGAATGCCACGCTCTTATCCGGCACAGTCCATGTGAGTGCAATGCTCTATTACGGGGAGA

SsBRV1 2273 TCAACGCTACGATTCTTTCTGGCACAGTACATGTCAGTGCTATGCTCTACTACGGTGAAA

BmBRV1-BdEW220 2324 AGCCTCGAACTGGAGCACTCAAATACATCTGGGATGAGAACCAGAGGGTATCGCTTGCCA

BmBRV1 2325 AGCCTCGAACTGGAGCACTCAAATACATCTGGGATGAGAACCAGAGGGTATCGCTTGCCA

SsBRV1 2333 AACCGAGAACAGGGGCACTCAAGTACATCTGGGAGGAGACTAAGCGCGTTAGCCTAGCCA

BmBRV1-BdEW220 2384 GGCAAAAATTCGTTACTGGCCCTCAATCTAAGGTCTTGAAGTTCCTATACTCTGGTAAAG

BmBRV1 2385 GGCAAAAATTCGTTACTGGTCCTCAATCTAAGGTCTTGAAGTTCCTGTACTCTGGTAAAG

SsBRV1 2393 GGCAGAAGTTTGTCACCGGACCCCAGTCTAAGGTGCTGAAGTTCTTGTATTCTGGGAAGG

BmBRV1-BdEW220 2444 CCAACATTTTTGATACCAACCCCACCGATTGGATGCATTATCTTGCTGACTTCAGCAAGC

BmBRV1 2445 CCAACATTTTTGATACCAACCCCACCGATTGGATGCACTATCTTGCTGACTTCAGCAAGC

SsBRV1 2453 CTAATATCTTCGACACCAACCCAACAGACTGGATGCACTACCTTGCTGACTTTAGCAAGC

BmBRV1-BdEW220 2504 GCCGTGACACTAGAAAGATTGACTCTTCTCTGGGTGTGCGGTCGACGAATACAGTTACCG

BmBRV1 2505 GCCGTGACACTAGAAAGATTGACTCTTCTCTGGGTGTGCGGTCGACGAATACAGTTACCG

SsBRV1 2513 GTCGCGATGCCAGGAAGATCGATTCATCTCTAGGCGTACGGTCGACCAATACAGTCACCG

BmBRV1-BdEW220 2564 GAGAGCCGCGTCTGATCTACCTTGGACTGCGACCCGCAATTCTTCAGTACGAGGAGAGGA

BmBRV1 2565 GGGAGCCGCGTCTGATCTACCTTGGACTACGACCCGCAATTCTTCAGTACGAGGAGAGGA

SsBRV1 2573 GAGAACCTAGATTGATCTACCTTGGGCTTAGACCAGCCATTGTTCAGTATGAGGAACGCA

BmBRV1-BdEW220 2624 TGGCCGAAGCGCAAGACGCATCTGGTATCGCATCTCACTACGAGCCAGCATTTGACTTTA

BmBRV1 2625 TGGCCGAAGCGCAAGACGCATCTGGTATCGCATCTCACTACGAGCCAGCATTTGACTTTA

SsBRV1 2633 TGTCTGAAGCCCAAGATGCTTCAGGTCTTGCATCCCACTACGAGCCTGCTTTCGATTTCA

BmBRV1-BdEW220 2684 ACAGCGCAATCTCTTTCTCCTCCGCTGTCATGAACCTAGGGGCGAAGCCGAAGGATGGAC

BmBRV1 2685 ACAGCGCCATTTCTTTCTCCTCCGCTGTCATGAACCTAGGGGCGAAGCCGAAGGATGGAC

SsBRV1 2693 ATAACGCAATCTCGTTCTCTTCAGCAGTAATGAACCTCGGCACCAAACCCAAGGACGGCC

BmBRV1-BdEW220 2744 GCCATGCAGATGATCCCGATACCCACGACGGAAACGCTCCCGTGCCTATGCCACGTAAGA

BmBRV1 2745 GCCATGCAGATGATCCCGATACCCACGACGGAAACGCTCCCGTGCCTATGCCACGTAAGA

SsBRV1 2753 GCCATTCTTCCGACCCCAGTACACACGATGGAAACGCACCCGTACCAATGCCAAGGAAGC

BmBRV1-BdEW220 2804 GGTCCAATAGCGTCTCGGCCCAACTGGGTGTAGACAAGATCTCGCCAGCAGGGCGCTTAT

BmBRV1 2805 GGTCCAATAGCGTCTCGGCCCAACTGGGTGTAGACAAGATCTCGCCAGCAGGGCGCTTAT

SsBRV1 2813 GTTCGAACAGTGTCTCTGCCCAACTCGGAGTAGACCAAATCTCACCCGCAGGGCGCTTCT

BmBRV1-BdEW220 2864 TCAGCGAGCTCTTCAAGGCAAAAT------CTAACAAACCTAAATCGCAAAGCGCAGACA

BmBRV1 2865 TCAGCGAGCTCTTCAAGGCCAAAC------CTAACAAACCTAAATCGCAAAGCGCAGATA

SsBRV1 2873 TCTCGGAAGTTTTCAAAGTTAAGCAACCCAAGAAGAAAGCTGACTCACA------AGAGC

BmBRV1-BdEW220 2918 GGCCAGATACAGGCAAATCTGAGAAGCTGTTTGAGCAAGGAGGTGTCGGTAGCGTACGCG

BmBRV1 2919 GGCCAGATACAGGCAAATCTGAGAAGCTGTTTGAGCAAGGAGGTGTCGGTAGCGTACGCG

SsBRV1 2927 AACCCGACAAGGGCGACTCAAAGAAGCTTTTCGATCAAGGTGGGGTTGGCAGTGTACGTG

BmBRV1-BdEW220 2978 CAACTTTAGTTCATGCTGCAAAAGACGCGCAACGCAATGGGCTTGCAGAGATCTTGCGAC

BmBRV1 2979 CAACTTTAGTTCATGCTGCAAAGGATGCGCAACGCAATGGGCTTGCAGAGATCTTGCGAC

SsBRV1 2987 GTACATTGATTCAACCCGCCAAAGATGCGAGATTCAATGGCTTAGCCGAAACGTTGCGCC

BmBRV1-BdEW220 3038 AGCTGAADGTCGAATCTTCATCACGTCTGGAAGATATTCCAGACCAACTCAAAAACCCCG

BmBRV1 3039 AGCTAAAGGTCGAATTTTCGTCACGTCTGGAAGATATTCCAGACCAACTCAAAAACCCCG

SsBRV1 3047 AATTGAAAGCCGAGTCTTCTTCGAGATTGGACGACATCCCCGAGCAACTCAAAAACCCCG

BmBRV1-BdEW220 3098 AGCACGCAAAGGGAAGTGAGATACTCGAAATCGGAGTGGAGGAAGCCTTCGATAACATTG

BmBRV1 3099 AGTACGCAAAGGGAAGTGAGATACTCGAAATCGGAGTGGAGGAAGCCTTCGATAACATTG

SsBRV1 3107 AGTACGCCAAGGGAAGCGAGATCCTCGAAATCGGCGTTGAAGAAGCTTTCGATAACATCG

BmBRV1-BdEW220 3158 AAAAACACTTCAACGCTCAGGATATTGAGTACACTTACGCTCCACTCGGAGCCCGAGACT

BmBRV1 3159 AAAAGCACTTCAACGCTCAGGATATTGAGTACACTTACGCTCCACTCGGAGCCCGAGACT

SsBRV1 3167 AGAAACACTTCAAAGCGCAAGATATCGAGTATACGTATGCTCCTCTCGGCGCGAAACAGT

BmBRV1-BdEW220 3218 ATCGGGAGTTCGGCTTGCATGCAAGCATCCAGGCTGTCAAGGGCGATGGTCGCTGTGGTG

BmBRV1 3219 ATCGGGAGTTCGGCTCGCATGCAAGCATCCAGGCTGTCAAGGGCGATGGTCGCTGTGGTG

SsBRV1 3227 ATCGTGAGCTCACGCTCCATGCTAAACTTCAGGCTGTTAAAGGAGATGGCCGCTGTGGAG

BmBRV1-BdEW220 3278 TTCGATCACTGCAAACAGCTTCAGTAGTCAACAACTTGAAGCCTTATTTAGAGCTTGACT

BmBRV1 3279 TTCGATCACTGCAAACAGCTTCAGTAGTCAACAACTTGAAGCCTTATTTAGAGCTTGACT

SsBRV1 3287 CTCGAGCTTTGCAGACTGCTTCTGTCGTCAACAACATCAAGCCTTACTTGGAGCTGAATG

BmBRV1-BdEW220 3338 CACTCTTTAAGACTGAGTCTCAGATCATGGGTCTCCAGACAACGACTACAGCACCAGCAA

BmBRV1 3339 CACTCTTTAAGACTGAGTCTCAGATCATGGGTCTCCAGACAACGACTACAGCACCAGCAA

SsBRV1 3347 CCCTCTTCAAGACTGAAGCCCGCATCATGGGGCTTCAGACTTCAGAGGTTGCACCCGCAA

BmBRV1-BdEW220 3398 CCCATATGGCTGATGACTATAGCCTTGCGAGCGTCGCAGCCGAGTACGGCTTCTCAGTAT

BmBRV1 3399 CCCATATGGCTGATGACTATAGCCTTGCGAGCGTCGCAGCCGAGTACGGCTTCTCAGTAT

SsBRV1 3407 CACATATGGCTGATGACTACAGCCTTGCAAGCCTTGCAAGCGAATACAATCTTGCAGTGT

BmBRV1-BdEW220 3458 GTATTGTCCATTACCATGGCAACATCAAGAGAAACCAGAAAGGCATCCGCTTTTACAAAC

BmBRV1 3459 GTATTGTCCATTACCATGGCAACATCAAGAGAAACCAGAAAGGTATCCGTTTTTACAAGC

SsBRV1 3467 GCATTGTGCACTACAGCGGGGACATTAAGCGAAACCAGAAAGGGCTTCGCTTCTATAGAC

BmBRV1-BdEW220 3518 CACGGAATGTTAAGAATCCCCGCGTACTTTACGTCCATTTAAAGGACTCCCACTACGATG

BmBRV1 3519 CACGAAATGTTAAGAATCCCCGCGTACTTTACGTCCACTTGAAGGACTCCCACTACGATG

SsBRV1 3527 CTCGAGGAGGACAGCGCTCTCAAGTACTGTTTGTTCACCTACAGGACTCGCACTACGAAG

BmBRV1-BdEW220 3578 CGTTAAAAGTTGACAACAACTTCAAGCCAGTTCTCGATGCTGAGAATGCATCGCAAGTGC

BmBRV1 3579 CGTTAAAAGTTGACAACAACCTCAAGCCAGTTCTCGGTGCTGAGAATGCATCGCAAGTGC

SsBRV1 3587 CTTACAAGTTTGAGAGTTCTTGGAAAGCACTTCTGGAAGCTGAAAATGCATCAGAGGTTT

BmBRV1-BdEW220 3638 TCGCCTGGTTGAAAGACAGTGCTGAGCTCGCACACTTGGGCGCATCAAATCAGAGTGAC-

BmBRV1 3639 TCGCCTGGTTGAAAGACAGTGCTGAGCTCGCACACTTGGGCGCATCAAACCAGAGTGAC-

SsBRV1 3647 CTGCATGGCTGACCGACCGCTTCGAAACCGCA---------GCACTAAATCGTGCTGTCC

BmBRV1-BdEW220 3697 --------GCTGACAGCGACTCTGACTCTGGACT-AGCCCGAAAGAGTAAGCCGCAGCAA

BmBRV1 3698 --------GCTGACAGCGACTCTGACTCTGGACT-AGCCCGAGAGAGTAAGCCGCAGCAA

SsBRV1 3698 TCGAAGCTGCCGAGAGCGACTCAGATTCTGGAATGAGTAAGAAGAAGTACGC----GAAG

BmBRV1-BdEW220 3748 TCTCTCGCTAAAAACTTGTCTTCTCTCAAGGATAGCGAGAGTGAGTCAGCATC-------

BmBRV1 3749 TCTCTCGCTAAAAACTTGTCTTCTCTCAAGGATAGCGAGAGTGAGTCAGCATC-------

SsBRV1 3754 TCCCTGGCCGCCAATTTGTCGCGTCAAGGAAGAAGCGAAAGTGATTCTGAATCAGAAGAT

BmBRV1-BdEW220 3801 --------TTCATCAGACGACGAATCTCCACCAATGCCAGCACCTAAGGCTGAGAAAACC

BmBRV1 3802 --------TTCATCAGACGATGAATCCCCACCAATGCCAGCACCTAAGGCTGAAAAAACC

SsBRV1 3814 GATAAGAGCGTGACGTATGACGAATCCTTCCCGGCTCTACCTACAGCAGCAGAGAAGATA

BmBRV1-BdEW220 3853 CAAGGTGGCGCTTCACGCAAGAAGCGGAACAACAGGAAGATAGCTAAACAGATGAACGCT

BmBRV1 3854 CAAGGTGGCGCTTCACGCAAGAAGCGGAGCAACAGGAAGACAGCTCAACAGATGAACGCT

SsBRV1 3874 AAAGGAGGTGCTTCCAAGAGGAAGCGTTCAAACCGTCGTATGACTAGGAGACTGAGCGAG

BmBRV1-BdEW220 3913 CAGAGGGAAGTTCAAGAAGAAAAAGTCTTCGAAGATGCACAGGTGTACAACCAGCGCATG

BmBRV1 3914 CAGAGGGAAGCTCAAGAAGAAAAAGTCTTCGAAGATGCACAGGTGTACAACCAGCGCATG

SsBRV1 3934 CAACAGAGACTCCAGGAGGAAGAGATCTTTTCTTCAGCTGAGGAATACAACCGCCAAAT-

BmBRV1-BdEW220 3973 TCAGAGCCGTCTTCAGGAGCGGATAGCGGCCCCTCCACTCCAGTAGACCGAAAAGCTTC-

BmBRV1 3974 TCAGAGCCGTCTTCAGGAGCGGATAGCGGCCCCTCCACCCCAGTAGACCGAAAAGCTCC-

SsBRV1 3993 -----------------------------------CCCTCCAACTGTCCCAGAATCTCCA

BmBRV1-BdEW220 4032 -----TCTGGCGGCAACCC---------GCACCGTGGTTGGCCTAGATACAGGCAAGACT

BmBRV1 4033 -----TCTGGCGGCAACCC---------GCACCGTGGTTGGCCTAGATACAGGCAAGACT

SsBRV1 4018 AGAAGCGTAAAGTCAACCCAGAGCTTTAGCTCGATGGTAGGCATTAACACGGGCAAGAGC

BmBRV1-BdEW220 4078 GTAGCCGAAACTTCTTTAGCCAGGACCACTACTACATCGGTTAGAGCTAACCGCAACCAC

BmBRV1 4079 GTAGCCGAAACTTCTTTAGCCAGGACCACTACTACACCGGTTAGAGCTAACCGCAATCAC

SsBRV1 4078 ATCCATGAGTCTT----------------TACCTCAAC-----GAATGAGCGCCAACAAA

BmBRV1-BdEW220 4138 CTCTACGCATTTGAGAAGGATGACGGAAACAGCTTAGCAATGGTCACCGCACTAAGAGAG

BmBRV1 4139 CTCTACGCATTTGAGAAGGATGACGGAAACAGCTTAGCAATGGTCACCGCACTAAGAGAG

SsBRV1 4117 CTTCTACCATCCCAACAAGACGAAGGTGAAAGGCAGGCAACGCTGAGTGCTATGAAAGAG

BmBRV1-BdEW220 4198 ACTTTCGAGCAACTGCAATTGGACAGCGAAGGTTCTCTTTCTGTAAACGAGGTAGAACTC

BmBRV1 4199 ACTTTCGAGCAACTGCAATTAGACAGCGAAGGTTCTCTTTCTGTAAACGAGGTAGAACTC

SsBRV1 4177 AGCTTTGAGCAGCTCGCACTCGATGGTAGTCTTTCGGATCCCGACATTGAAGTTGAGAGA

BmBRV1-BdEW220 4258 CGCATCGTCCGGGAGTGCCGGCGCTTTGTCCGAGAAGACCAATGGGCAGGAACTGTATCA

BmBRV1 4259 CGCATCGTCCGGGAGTGCCGGCGCTTTGTCCGAGAAGACCAATGGGCAGGAACTGTATCA

SsBRV1 4237 CGCATCACTCGTGAGTGCAAGCGTTTCGTGCGTGATGAACAATGGTCAGGAACGGTATCT

BmBRV1-BdEW220 4318 AAGTACCGTATGACAGAGTTCATCACACAGATCCCCCACTCAATTAGGGGTGCCACAAGC

BmBRV1 4319 AAGTACCGTATGACAGAGTTCATCACACAGATCCCCCACTCGATTAGGGGTGCCACAAGC

SsBRV1 4297 AAGTACCGCATGACTGAGTTTGTGACACAGCTGCCTTACTCACTCAGGGATGCATCTTCT

BmBRV1-BdEW220 4378 ATCGGCCGCTTCCGACTCTGGAAGTACCTCGAGACTCTACCTGGTGTTGATACCAAAGTA

BmBRV1 4379 ATAGGCCGCTTCCGACTCTGGAAGTACCTCGAGACTCTACCTGGTGTTGATACCAAAGTA

SsBRV1 4357 ATCTCGAGACCCAGGTTGATCCAGTATATCAAGTCGTTTCCTTCGGTCGGTGATAAGATA

BmBRV1-BdEW220 4438 ATCAGAGCAGTCTCTTCACTTGCGCTAGCTGCAGGGACTACCTGGGATTACATGGCACAA

BmBRV1 4439 ATCAGAGCAGTCTCTTCACTTGCGCTAGCTGCAGGGACTACCTGGGATTACATGGCACAA

SsBRV1 4417 TTCGAAGCTGTAGCAACTTTGGCTATGGCTGCAGGTGTGACATACGAATATATGACGCAA

BmBRV1-BdEW220 4498 GACCACAGCGTAGGAGAAGATGAGCTGATGTACTACATTATCCATGCACAGCTTTCTGCA

BmBRV1 4499 GACCACAGCGTAGGAGAAGATGAGCTAATGTATTACATTATCCATGCACAGCTTTCTGCA

SsBRV1 4477 CCTAAGTCCGTAGGTGACGACGAACTGATGTATTATTTGATCAAGGCACAGTTGCCTGCT

BmBRV1-BdEW220 4558 GTTACGATAGACTTCAATGCTGTCTACTACGGACTCCCGAACACCAAGAAGGTAGTCGGA

BmBRV1 4559 GTTACGATAGACTTCAATGCTGTCTACTACGGCCTCCCGAACACCAAGAAGGTAGTCGGA

SsBRV1 4537 GTCGCTATAGAATATAACGCTGTGTACGCTGGAATTCCATTCACAAAAAAGATTGTAGGG

BmBRV1-BdEW220 4618 TATACCACCTACGAGACAGGGTGTCGCCACAACATTCACGCTGAAAACACCGTCGTATTC

BmBRV1 4619 TATACCACCTACGAGACAGGGTGTCGCCACAACATTCACGCTGAAAACACCGTCGTATTC

SsBRV1 4597 TATACTACCTACGACACCGGAAAACATTCTGGCCCAGATGCTCACCACACTCTGGTCTTT

BmBRV1-BdEW220 4678 ATTCGGCAAGAAGGGCATCTCTGTGCCCCATACATCACGAGGAAGTCTGCAGACTCTCTC

BmBRV1 4679 ATTCGGCAAGAAGGGCATCTCTGTGCCCCATACATCACGAGGAAGTCTGCAGACTCTCTC

SsBRV1 4657 GTCAGCCAGCAAGGACACTTGTGTGCCCCATATCTGACGCGAGAATCTGCCGACTCCTTG

BmBRV1-BdEW220 4738 ATCCTAGAGATGAAAGACATCAGCAACTACTCGAAGCTCCACAACACTATGGATGATCCT

BmBRV1 4739 ATCCTAGAGATGAAAGACATCAGCAACTACTCGAAGCTCCACAGCACTATGGATGATCCT

SsBRV1 4717 ATCCTAGAGATGAAGGATATCAGTAACTACAGCAAGGTGGTCCACTCAGGACAAGGTTTT

BmBRV1-BdEW220 4798 ACCGAACCGTCCTTCCTAGCTCCGACAAGAAGCAAAGAATTGTGGGTGCAGTCCGTGAAT

BmBRV1 4799 ACCGAACCGTCTTTCCTAGCTCCGACAAGAAGCAAAGAATTGTGGGTGCAGTCCGTAAAT

SsBRV1 4777 GATGGAGTGTCAAGCTTATCCCCGACAAGAACGCGAGAACTTTGGATCCAAGCTGTTAAC

BmBRV1-BdEW220 4858 ACTGAGATCGCCAAGATGGGTGCCACGTCAAGAAGGTTTTCTAACCTCAAGTGCGATCAA

BmBRV1 4859 ACTGAAATCGCCAAGATGGGTGCCACGTCAAGAAGGTTTTCTAACCTCAAGTGCGATCAA

SsBRV1 4837 GTCGAGATTGCAAAGATGGGCGCTACTTCACGAAAACTGAGTGGCATTGCTTGCGACCCC

BmBRV1-BdEW220 4918 GCATATTGGGAGAAAGCTTTGTCCAGTCGTACATGCATTGGTTCAGATCCAGACGACACA

BmBRV1 4919 GCATATTGGGAGAAAGCTTTGCCCAGTCGTACATGCATTGGTTCAGATCCAGACGACACA

SsBRV1 4897 GTATACTGGGCAAAGGCATTAGAGAGCATAACTTGTGTCGGCTCAGACCCTGACGATATT

BmBRV1-BdEW220 4978 GCTGAGAACAACATCGAACTCCTGTCATGTTTTGCTGACTACCTTGGTTGCCCTGACGAC

BmBRV1 4979 GCTGAGAACAACATCGAACTCCTGTCATGTTTTGCTGACTACCTTGGTTGCCCTGACGAC

SsBRV1 4957 GCCGAAAACAACATTGAGTTATTATCTTGCTTCGCAGACTATCTGGGATGCCCCGATGAC

BmBRV1-BdEW220 5038 GTCTCATTCTTCAGAACAGAGGCAATCGATTGGCTCGTACGAGTCATCGGCTGGGTACCA

BmBRV1 5039 GTCTCATTCTTCAGAACAGAGGCAATCGATTGGCTCGTACGAGTCATCGGCTGGGTACCA

SsBRV1 5017 GTATCTTTTTTTCGAACGGAAGCAGTAGACTGGCTCATTCGAGTTCTCGGGTGGGTACCC

BmBRV1-BdEW220 5098 GCAGACCAGTCTTGGTTCGGAGCCGAGACCGTCATGCTCCTCGCGCTGCACAGAGACTTC

BmBRV1 5099 GCAGACCAGTCTTGGTTTGGAGCCGAGACCGTCATGCTCCTCGCGTTGCACAGAGACTTC

SsBRV1 5077 GACGACCAAGCATGGTTCGGAATGGAGACAGTAATGCTTCTTGCACTACACAGAGATTTC

BmBRV1-BdEW220 5158 CGAGTTTGGGTTGTCGAAGACACACCTGACCTCTCCATCCACAACCTCGTGGAGCATAAG

BmBRV1 5159 CGAGTTTGGGTTGTCGAAGACACACCTGACCTCTCCATCCACAACCTCGTGGAGCATAAG

SsBRV1 5137 CGAGTTTGGGTTATTGAAGAAACCGGAGACACACAAGTACACAACATGACAGAGCATAAG

BmBRV1-BdEW220 5218 TCCTCGAAAACGCAGAGTACACTCCCACTGATTATGAGTGTCCAGTCTGGCAAGCTTAGG

BmBRV1 5219 TCCTCTAAAACGCAGAGTACACTCCCACTGATTATGAGTGTCCAGTCTGGTAAGCTTAGG

SsBRV1 5197 TTTGCAAGGACTCAGAATACACTCCCGATCATCATGAGTGAACAAGCTGGAAGACTGAGA

BmBRV1-BdEW220 5278 ATGCACCAGCTGAACTTACCCGCACGAGCAGGCTCTTCAATGGAGAACCTAGCACTAGCA

BmBRV1 5279 ATGCACCAGCTGAACTTACCCGCACGAGCAGGCTCTTCAATGGAGAACCTAGCACTAGCA

SsBRV1 5257 ATGCACCAGCTAAGACTCCCAGCAAGAGCAGGTTCTTCGCTCGAAAATCTCGCATTGGCT

BmBRV1-BdEW220 5338 TGCTTACAGCATAGACACAATGGCACTTACCCAGAGCCGAGTGAGCCAACCCCCACCCTC

BmBRV1 5339 TGCTTACAGCATAGACACAATGGCACTTACCCAGAGCCGAGTGAGCCAACCCCCACCCTC

SsBRV1 5317 TGCAAACAACATAGGCGAGATGGCAAATACCCAGAGCCCAGCGAACCGACACCTACTTTA

BmBRV1-BdEW220 5398 CCTACACCACAAAGACCAGACGACCTGAAACTCAAGAACCCCATGCAAGGGCACCTCCTT

BmBRV1 5399 CCTACACCACAAAGACCAGACGACCTGAAACTCAAGAACCCCATGCAAGGGCACCTCCTA

SsBRV1 5377 CCAACACCCCAAAGACCTAGTGATCTGAAACTCAAGCATTCTATGCAAGGGCACATACTC

BmBRV1-BdEW220 5458 GGCTGGGTTATAGCAGGTAGTACTCTTATAGTTACTGTAGGCTTAGGTGTCGTTTCTTAT

BmBRV1 5459 GCCTGGGTCATAGCAGGTAGTACTCTTATAGTTACTGTAGGCTTAGGTGTCGTTTCTTAT

SsBRV1 5437 GGTCTAGTGATAGCAGGAACCACATTGGTTGTGACGGCTGGTGTCGCGGTAGTCTCTTAC

BmBRV1-BdEW220 5518 CGCCACAGGAAGCAGATTAATGGGTTAGTCAAGAATGTGCGAGATATGTGGAAGGGCACG

BmBRV1 5519 CGCCACCGGAAGCAGATCAATGGGTTAGTCAAGAATGTGCGAGATATGTGGAAGGGCACG

SsBRV1 5497 AGGAAACGTAAGAGTATCAGCGGACTAGTAAAGAATGTGAGGGATAGATGGAAGGGGGTA

BmBRV1-BdEW220 5578 TCTGTCTACACAGCTAGTATTAGAGCATTGGAAGAGGTCCAGGACACAGAGCCGTTGCTC

BmBRV1 5579 TCTGTCTACACAGCTAGTATTAGAGCATTGGAAGAGGTCCAGGACACAGAGCCGTTGCTC

SsBRV1 5557 TCTGCCTACACAGCAAGCATCCGAACTCTCCAAGAGATTGCAGATACAGAACCACTTCTC

BmBRV1-BdEW220 5638 GGAGATCAAAACACACCATTCACGAAGGACGCGCAGCCTGGCCCTCTCTCACCGTACTAC

BmBRV1 5639 GGAGATCAGAACACACCATTCACGAAGGACGCGCAGCCTGGCCCTCTCTCACCGTACTAC

SsBRV1 5617 GGCAATCATGACACTGCTTTCACGAGTGATGCGCAGCCAGGGCCACTCTCACCGAACTTT

BmBRV1-BdEW220 5698 GAGGATTTCCAACAAGCCATCGGAGTCAAAGACCATCTGTCGGACGCCGGCTCTGTTCGC

BmBRV1 5699 GAGGATTTCCAACAAGCCATCGGAGTCAAAGACCACTTGTCGGATGCCGGCTCTGTTCGC

SsBRV1 5677 GAAGATTTTCAGCAAGCATTAGGGTTGAAAGATCATCTCTCTGATGTCGGCTCAGTACGC

BmBRV1-BdEW220 5758 AGCTTGCGAACGGTGCCAATAGGCGACCATAGTGTGTTCGCTACAGGACGCTGGCCAGGC

BmBRV1 5759 AGCTTGCGAACGGTACCAATAGGCGACCATAGTGTGTTCGCTACAGGACGCTGGCCAGGC

SsBRV1 5737 AGTTTACGGACAGTACCTCTGGGAGGGAATACCGCCTTCGCAACGGGACGTTGGCCAGGA

BmBRV1-BdEW220 5818 ATTGTCTTGTTCAAAAGATGGTGGGGTAATGCAGGTGAAGCAGTCCACAACTAAGTTATA

BmBRV1 5819 ATTGTCTTGTTCAAAAGATGGTGGGGTAATGCAGGAGAAGCAGTCCACAACTAAGTTATA

SsBRV1 5797 ATTGTACTCTTCAAGAGATGGTGGGGTACAGCAGGTGAAACAGTCCAAAATTAAGTTACA

BmBRV1-BdEW220 5878 GCACGCAGCCGTAACTTCCGGGAATAGCCGGACAAAATAGCAGAAACATAGTTTTCATAC

BmBRV1 5879 GCACGCAGCCGTAACTTCCGGGAATAGCCGGACAAAATAGCAGAAACATAGTTTTCATAC

SsBRV1 5857 GCACGCAGCAGTAACTTCCGGGAATAACCGGACATATTAGCAGAAACACAAGTTTCATAC

BmBRV1-BdEW220 5938 GAGTCAAGGGTTAGTCCCAAAATTAAGTCCCAAGAGACCTACAAATAGT

BmBRV1 5939 GAGTCAAGGGTTAGTCCCAAAATTAAGTCCCAAGAGACCTACAAATAGC

SsBRV1 5917 GAGTCAAGGGTTAGTCCCAAATTTAGGTCCCCAGCGACCTACAAATAGC
