# Supplementary material for: Characterization of a Botybirnavirus Conferring Hypovirulence in the Phytopathogenic Fungus Botryosphaeria dothidea
Source: Viruses. 2019 Mar 17;11(3):266. doi: 10.3390/v11030266 (PMC6466033; doi:10.3390/v11030266)
Supplement: Supplementary file 1 [file viruses-11-00266-s001.zip › viruses-449497-supplementary/Manuscript supplementary Figure 4.docx]

**Supplementary**

**Figure S4.** Multiple-sequence alignments of the sequences of P2 encoded by BmBRV1-BdEW220, BmBRV1 and SsBRV1. Abbreviations: BmBRV1-BdEW220, Bipolaris maydis botybirnavirus 1 strain BdEW220; BmBRV1, Bipolaris maydis botybirnavirus 1; SsBRV1, Sclerotinia sclerotiorum botybirnavirus.

BmBRV1-BdEW220 1 MSYRSNSVSLHSVPSKQLLGCTSMLFVEKTRGGVNLKKKMPTRVEDAGIRASGKLGGKET

BmBRV1 1 MSYRSNSVSLHSVHSKQLLGCTSMLFVEKTRGGVNLKKKMPTRVEDAGIRASGKLGGKET

SsBRV1 1 MSYRSNSISLNSVRSKHMRGVTFVLPVEKTRGGISLKKKMPTRVEDAGIRASEQLGGKET

BmBRV1-BdEW220 61 SATASEASPLGGLFDAVSALSMESADPQFGNPSNVLEGESYSELCLTQSILSRMPDINFK

BmBRV1 61 SATASEASPLGGLFDAVSALSMESADPQFGNPSNVLEGESYSELCLTQSILSRMPDINFK

SsBRV1 61 SATASEASPLGGLFDAVTSLATEVADPQFGTPSTILEGESYSELCLTQSILSRMPDINFK

BmBRV1-BdEW220 121 NPFLVQAPSAKDQVVYMAWGYKLKSLPSSQKGSAYADKYTVKLWAKTNEVRVQYPVIATS

BmBRV1 121 NPFLVQAPSAKDQVVYMAWGYKLKSLPSSQKGSAYADKYAVKLWAKTNEVRVQYPVIATS

SsBRV1 121 NPFLVQAPTAKYQTVYMAWGYKLKSLPSSQKGSAYADKYGVKLWAKTKEVRVNYPVIATS

BmBRV1-BdEW220 181 VGYRNKITTEYEQRATGNLVTTQRVDSREFQASFDEMKGFPIQFRLGGSAKSVDGDNHIA

BmBRV1 181 VGYRNKITTEYEQRATGNLVTTQRVDSREFQASFDEMKGFPIQFRLGGSAKSVDGDNHIA

SsBRV1 181 TGYRNRITTEYEQRATGNLVTTQRVDSREFQARFDEMKGFPIEFRLGGTAKSVDGDNHVA

BmBRV1-BdEW220 241 WLVIAILRLFALKQAQETNSRGHITMSTAIASSFSINLEDMVQARSSASHSVATALANAW

BmBRV1 241 WLVIAILRLFALKQAQETNSRGHITMSTAIASSLSINLEDMVQARSSASHSVATALANAW

SsBRV1 241 WLTIAILRLLALKQAQETNSRGHIEMTSGIANQFGINLEDMVQARSSSSHSVAPALANAW

BmBRV1-BdEW220 301 FGAAKPGGRPGAVEAVPDKMFEMVLPSTAADVSEVVYLAYLSGMLDNSMRWRNGDEELGI

BmBRV1 301 FGAAKPGGRPGAVEAVPDKMFEMVLPSTAADVSEVVYLAYLSGMLDDSMRWRNGDEELGI

SsBRV1 301 FGSAKSGGKPGAVEAVPDRVFEMVLPSNAADASEVVYLAYLSGMLDDSMRWRNGDEELGI

BmBRV1-BdEW220 361 QPMFSSIRSTATERMKIPLVNASRAIPMADLRGYDSDLELGRAEAVFNSYVRRHELQSQV

BmBRV1 361 QPMFSSIRSSATERMKIPLVNASRAIPMADLRGYDSDLELGRAEAVFNSYVRRHELQSQV

SsBRV1 361 QPMYSAIRSSATERMKIPFVNASRAISMNDLKKFNADIELGRAEAVFNSYVRRHELQSQV

BmBRV1-BdEW220 421 NVARRIALLAMLDTHSSKSSRMLLGLPKPCHVVEYDLWVDPKHFSTSPLSLIATSESAGL

BmBRV1 421 NVARRVALLAMLDTHSSKSSRMLLGLPKPCHVVEYDLWVDPKHFSTSPLSLIATSESAGL

SsBRV1 421 NVARRIALLAMLDTHSNKGSRMLLGLPKPCHVLEYDLWVDPKHYSTSPLNLIATSESAGL

BmBRV1-BdEW220 481 LMALSQMQATMRNDILVMKLIDHLESKGTPVLSSLAYESLRDWIYDAMPSGFSSWSRGWL

BmBRV1 481 LMALSQMQATMRNDILVMKLIDHLESKGTPVLSSLAYESLRDWIYDAMPSGFSSWSRGWL

SsBRV1 481 LMALSQMQATMRNDILVMKLIDHLESRGTPVLSSLAYESLRDWIYDAMPSGFSSWSRGWL

BmBRV1-BdEW220 541 STVTGTSPPEIRRLANDSVHGFWTGLNATLLSGTVHVSAMLYYGEKPRTGALKYIWDENQ

BmBRV1 541 STVTGTSPPEIRRLANDSVHGFWTGLNATLLSGTVHVSAMLYYGEKPRTGALKYIWDENQ

SsBRV1 541 NTVTGGSPPEIRRLANDSVHGFWTGLNATILSGTVHVSAMLYYGEKPRTGALKYIWEETK

BmBRV1-BdEW220 601 RVSLARQKFVTGPQSKVLKFLYSGKANIFDTNPTDWMHYLADFSKRRDTRKIDSSLGVRS

BmBRV1 601 RVSLARQKFVTGPQSKVLKFLYSGKANIFDTNPTDWMHYLADFSKRRDTRKIDSSLGVRS

SsBRV1 601 RVSLARQKFVTGPQSKVLKFLYSGKANIFDTNPTDWMHYLADFSKRRDARKIDSSLGVRS

BmBRV1-BdEW220 661 TNTVTGEPRLIYLGLRPAILQYEERMAEAQDASGIASHYEPAFDFNSAISFSSAVMNLGA

BmBRV1 661 TNTVTGEPRLIYLGLRPAILQYEERMAEAQDASGIASHYEPAFDFNSAISFSSAVMNLGA

SsBRV1 661 TNTVTGEPRLIYLGLRPAIVQYEERMSEAQDASGLASHYEPAFDFNNAISFSSAVMNLGT

BmBRV1-BdEW220 721 KPKDGRHADDPDTHDGNAPVPMPRKRSNSVSAQLGVDKISPAGRLFSELFKAKSNKPKSQ

BmBRV1 721 KPKDGRHADDPDTHDGNAPVPMPRKRSNSVSAQLGVDKISPAGRLFSELFKAKPNKPKSQ

SsBRV1 721 KPKDGRHSSDPSTHDGNAPVPMPRKRSNSVSAQLGVDQISPAGRFFSEVFKVKQPKKKAD

BmBRV1-BdEW220 781 SADRPDTGKSEKLFEQGGVGSVRATLVHAAKDAQRNGLAEILRQLXVESSSRLEDIPDQL

BmBRV1 781 SADRPDTGKSEKLFEQGGVGSVRATLVHAAKDAQRNGLAEILRQLKVEFSSRLEDIPDQL

SsBRV1 781 SQEQPDKGDSKKLFDQGGVGSVRGTLIQPAKDARFNGLAETLRQLKAESSSRLDDIPEQL

BmBRV1-BdEW220 841 KNPEHAKGSEILEIGVEEAFDNIEKHFNAQDIEYXYAPLGARDYREFGLHASIQAVKGDG

BmBRV1 841 KNPEYAKGSEILEIGVEEAFDNIEKHFNAQDIEYTYAPLGARDYREFGSHASIQAVKGDG

SsBRV1 841 KNPEYAKGSEILEIGVEEAFDNIEKHFKAQDIEYTYAPLGAKQYRELTLHAKLQAVKGDG

BmBRV1-BdEW220 901 RCGVRSLQTASVVNNLKXYLELDSLFKTESQIMGLQTTTTXPATHMADDYSLASVAAEYG

BmBRV1 901 RCGVRSLQTASVVNNLKPYLELDSLFKTESQIMGLQTTTTAPATHMADDYSLASVAAEYG

SsBRV1 901 RCGARALQTASVVNNIKPYLELNALFKTEARIMGLQTSEVAPATHMADDYSLASLASEYN

BmBRV1-BdEW220 961 FSVCIVHYHGNIKRNQKGIRFYKPRNVKNPRVLYVHLKDSHYDALKVDNNFKPVLDAENA

BmBRV1 961 FSVCIVHYHGNIKRNQKGIRFYKPRNVKNPRVLYVHLKDSHYDALKVDNNLKPVLGAENA

SsBRV1 961 LAVCIVHYSGDIKRNQKGLRFYRPRGGQRSQVLFVHLQDSHYEAYKFESSWKALLEAENA

BmBRV1-BdEW220 1021 SQVLAWLKDSAELAHLGASNQSDADSDSDSGLARKSKPQQSLAKNLSSLKDSESESASSS

BmBRV1 1021 SQVLAWLKDSAELAHLGASNQSDADSDSDSGLARESKPQQSLAKNLSSLKDSESESASSS

SsBRV1 1021 SEVSAWLTDRFETAALNRAVLEAAESDSDSGMSKK-KYAKSLAANLSRQGRSESDSESED

BmBRV1-BdEW220 1081 D-----DESPPMPAPKAEKTQGGASRKKRNNRKIAXQMNAQREVQEEKVFEDAQVYNQRM

BmBRV1 1081 D-----DESPPMPAPKAEKTQGGASRKKRSNRKTAQQMNAQREAQEEKVFEDAQVYNQRM

SsBRV1 1080 DKSVTYDESFPALPTAAEKIKGGASKRKRSNRRMTRRLSEQQRLQEEEIFSSAEEYNRQI

BmBRV1-BdEW220 1136 SEPSSGADSGPSTPVDRKASLAATRTVVGLDTGKTVAETSLARTTTTSVRANRNHLYAFE

BmBRV1 1136 SEPSSGADSGPSTPVDRKAPLAATRTVVGLDTGKTVAETSLARTTTTPVRANRNHLYAFE

SsBRV1 1140 ------PPTVPESPRSVKSTQSFS-SMVGINTGKSIHE-------SLPQRMSANKLLPSQ

BmBRV1-BdEW220 1196 KDDGNSLAMVTALRETFEQLQLDSEGSLSVNEVELRIVRECRRFVREDQWAGTVSKYRMT

BmBRV1 1196 KDDGNSLAMVTALRETFEQLQLDSEGSLSVNEVELRIVRECRRFVREDQWAGTVSKYRMT

SsBRV1 1186 QDEGERQATLSAMKESFEQLALDGSLSDPDIEVERRITRECKRFVRDEQWSGTVSKYRMT

BmBRV1-BdEW220 1256 EFITQIPHSIRGATSIGRFRLWKYLETLPGVDTKVIRAVSSLALAAGTTWDYMAQDHSVG

BmBRV1 1256 EFITQIPHSIRGATSIGRFRLWKYLETLPGVDTKVIRAVSSLALAAGTTWDYMAQDHSVG

SsBRV1 1246 EFVTQLPYSLRDASSISRPRLIQYIKSFPSVGDKIFEAVATLAMAAGVTYEYMTQPKSVG

BmBRV1-BdEW220 1316 EDELMYYIIHAQLSAVTIDFNAVYYGLPNTKKVVGYTTYETGCRHNIHAENTVVFIRQEG

BmBRV1 1316 EDELMYYIIHAQLSAVTIDFNAVYYGLPNTKKVVGYTTYETGCRHNIHAENTVVFIRQEG

SsBRV1 1306 DDELMYYLIKAQLPAVAIEYNAVYAGIPFTKKIVGYTTYDTGKHSGPDAHHTLVFVSQQG

BmBRV1-BdEW220 1376 HLCAPYITRKSADSLILEMKDISNYSKLHNTMDDPTEPSFLAPTRSKELWVQSVNTEIAK

BmBRV1 1376 HLCAPYITRKSADSLILEMKDISNYSKLHSTMDDPTEPSFLAPTRSKELWVQSVNTEIAK

SsBRV1 1366 HLCAPYLTRESADSLILEMKDISNYSKVVHSGQGFDGVSSLSPTRTRELWIQAVNVEIAK

BmBRV1-BdEW220 1436 MGATSRRFSNLKCDQAYWEKALSSRTCIGSDPDDTAENNIELLSCFADYLGCPDDVSFFR

BmBRV1 1436 MGATSRRFSNLKCDQAYWEKALPSRTCIGSDPDDTAENNIELLSCFADYLGCPDDVSFFR

SsBRV1 1426 MGATSRKLSGIACDPVYWAKALESITCVGSDPDDIAENNIELLSCFADYLGCPDDVSFFR

BmBRV1-BdEW220 1496 TEAIDWLVRVIGWVPADQSWFGAETVMLLALHRDFRVWVVEDTPDLSIHNLVEHKSSKTQ

BmBRV1 1496 TEAIDWLVRVIGWVPADQSWFGAETVMLLALHRDFRVWVVEDTPDLSIHNLVEHKSSKTQ

SsBRV1 1486 TEAVDWLIRVLGWVPDDQAWFGMETVMLLALHRDFRVWVIEETGDTQVHNMTEHKFARTQ

BmBRV1-BdEW220 1556 STLPLIMSVQSGKLRMHQLNLPARAGSSMENLALACLQHRHNGTYPEPSEPTPTLPTPQR

BmBRV1 1556 STLPLIMSVQSGKLRMHQLNLPARAGSSMENLALACLQHRHNGTYPEPSEPTPTLPTPQR

SsBRV1 1546 NTLPIIMSEQAGRLRMHQLRLPARAGSSLENLALACKQHRRDGKYPEPSEPTPTLPTPQR

BmBRV1-BdEW220 1616 PDDLKLKNPMQGHLLGWVIAGSTLIVTVGLGVVSYRHRKQINGLVKNVRDMWKGTSVYTA

BmBRV1 1616 PDDLKLKNPMQGHLLAWVIAGSTLIVTVGLGVVSYRHRKQINGLVKNVRDMWKGTSVYTA

SsBRV1 1606 PSDLKLKHSMQGHILGLVIAGTTLVVTAGVAVVSYRKRKSISGLVKNVRDRWKGVSAYTA

BmBRV1-BdEW220 1676 SIRALEEVQDTEPLLGDQNTPFTKDAQPGPLSPYYEDFQQAIGVKDHLSDAGSVRSLRTV

BmBRV1 1676 SIRALEEVQDTEPLLGDQNTPFTKDAQPGPLSPYYEDFQQAIGVKDHLSDAGSVRSLRTV

SsBRV1 1666 SIRTLQEIADTEPLLGNHDTAFTSDAQPGPLSPNFEDFQQALGLKDHLSDVGSVRSLRTV

BmBRV1-BdEW220 1736 PIGDHSVFATGRWPGIVLFKRWWGNAGEAVHN

BmBRV1 1736 PIGDHSVFATGRWPGIVLFKRWWGNAGEAVHN

SsBRV1 1726 PLGGNTAFATGRWPGIVLFKRWWGTAGETVQN
