# Supplementary material for: Characterization of a Botybirnavirus Conferring Hypovirulence in the Phytopathogenic Fungus Botryosphaeria dothidea
Source: Viruses. 2019 Mar 17;11(3):266. doi: 10.3390/v11030266 (PMC6466033; doi:10.3390/v11030266)
Supplement: Supplementary file 1 [file viruses-11-00266-s001.zip › viruses-449497-supplementary/Manuscript supplementary Table 1.docx]

**Supplementary**

**Table S1.** PMF-MS analysis of p110 encoded by ORF2 of Bipolaris maydis botybirnavirus 1 strain BdEW220.

| **Amino acid**  **position** | **Calculated**  **Mass** | **Observed**  **Mass** | **Mr(expt)** | **± delta** | **Amino acid sequence** | **Ions score** |
| --- | --- | --- | --- | --- | --- | --- |
| 132–142 | 1358.6329 | 1359.5201 | 1358.5128 | +0.1201 | DQVVYMAWGYK | 80 |
| 172–184 | 1451.7773 | 1452.6627 | 1451.6554 | +0.1219 | VQYPVIATSVGYR | 97 |
| 187–194 | 1038.4982 | 1039.4114 | 1038.4041 | +0.0941 | ITTEYEQR | 56 |
| 195–204 | 1059.5673 | 1060.4958 | 1059.4885 | +0.0788 | ATGNLVTTQR | 45 |
| 209–218 | 1230.5227 | 1231.4330 | 1230.4257 | +0.0970 | EFQASFDEMK | 71 |
| 209–225 | 2075.9775 | 2076.8340 | 2075.8267 | +0.1508 | EFQASFDEMKGFPIQFR | 117 |
| 353–368 | 1791.8461 | 1792.7037 | 1791.6964 | +0.1497 | NGDEELGIQPMFSSIR | 106 |
| 403–412 | 1154.5720 | 1155.4711 | 1154.4638 | +0.1082 | AEAVFNSYVR | 74 |
| 413–424 | 1435.7644 | 1436.6418 | 1435.6345 | +0.1299 | RHELQSQVNVAR | 71 |
| 538–552 | 1599.8257 | 1600.6998 | 1599.6925 | +0.1332 | GWLSTVTGTSPPEIR | 124 |
| 594–601 | 1122.5094 | 1123.4069 | 1122.3996 | +0.1098 | YIWDENQR | 54 |
| 626–645 | 2385.0736 | 2385.9099 | 2384.9026 | +0.1710 | ANIFDTNPTDWMHYLADFSK | 95 |
| 670–685 | 1946.0989 | 1946.9613 | 1945.9540 | +0.1449 | LIYLGLRPAILQYEER | 52 |
