# Supplementary material for: Characterization of a Botybirnavirus Conferring Hypovirulence in the Phytopathogenic Fungus Botryosphaeria dothidea
Source: Viruses. 2019 Mar 17;11(3):266. doi: 10.3390/v11030266 (PMC6466033; doi:10.3390/v11030266)
Supplement: Supplementary file 1 [file viruses-11-00266-s001.zip › viruses-449497-supplementary/Manuscript supplementary Table 2.docx]

**Supplementary**

**Table S2.** PMF-MS analysis of p90 encoded by ORF1 of Bipolaris maydis botybirnavirus 1 strain BdEW220.

| **Amino acid**  **position** | **Calculated**  **Mass** | **Observed**  **Mass** | **± delta** | **Amino acid Sequence** | **Ions score** |
| --- | --- | --- | --- | --- | --- |
| 323–333 | 1333.6514 | 1334.5547 | +0.1040 | SISPQFEELER | 79 |
| 383–409 | 2967.4457 | 2968.2717 | +0.1813 | SEIIPVSEDTIMYDTLAGLSIEGQMVR | 20 |
| 410–429 | 2244.0481 | 2244.9229 | +0.1325 | LNTTFNGNMVTDLYNSIGDR | 147 |
| 539–555 | 1880.8792 | 1881.7472 | +0.1393 | GVATEDEEIAVFSPFDR | 114 |
| 539–563 | 2857.3195 | 2858.1460 | +0.1808 | GVATEDEEIAVFSPFDRFHTDSNFK | 94 |
| 597–611 | 1851.8904 | 1852.7474 | +0.1503 | YVNQNDLWDQFAIAR | 127 |
| 751–762 | 1389.7041 | 1390.6078 | +0.1036 | AISFGWESQPIR | 76 |
| 845–865 | 2312.1397 | 2312.9832 | +0.1638 | SQLEHLEGTPTIWNTSTAATR | 142 |
