# Supplementary material for: Characterization of a Botybirnavirus Conferring Hypovirulence in the Phytopathogenic Fungus Botryosphaeria dothidea
Source: Viruses. 2019 Mar 17;11(3):266. doi: 10.3390/v11030266 (PMC6466033; doi:10.3390/v11030266)
Supplement: Supplementary file 1 [file viruses-11-00266-s001.zip › viruses-449497-supplementary/Manuscript supplementary Table 3.docx]

**Supplementary**

**Table S3.** PMF-MS analysis of p80 encoded by ORF1 of Bipolaris maydis botybirnavirus 1 strain BdEW220.

| **Amino acid**  **position** | **Calculated**  **Mass** | **Observed** **Mass** | **± delta** | **Amino acid Sequence** | **Ions score** |
| --- | --- | --- | --- | --- | --- |
| 383–409 | 2966.4617 | 2967.2769 | +0.1921 | SEIIPVSEDTIMYDTLAGLSIEGQMVR | 209 |
| 410–429 | 2244.0481 | 2244.8857 | +0.1697 | LNTTFNGNMVTDLYNSIGDR | 112 |
| 477–498 | 2326.2128 | 2327.0364 | +0.1837 | VEGDTLNANILLDNVVANSVTR | 218 |
| 507–535 | 3182.5311 | 3183.3569 | +0.1815 | SKPTNAVMLPHGSNDLDVETMLYLMGHGR | 117 |
| 539–555 | 1880.8792 | 1881.7188 | +0.1677 | GVATEDEEIAVFSPFDR | 134 |
| 539–563 | 2857.3195 | 2858.1326 | +0.1942 | GVATEDEEIAVFSPFDRFHTDSNFK | 102 |
| 597–611 | 1851.8904 | 1852.7363 | +0.1614 | YVNQNDLWDQFAIAR | 125 |
| 622–636 | 1631.8420 | 1632.6949 | +0.1544 | SFSASVGLPKPYHSR | 116 |
| 751–762 | 1389.7041 | 1390.5940 | +0.1174 | AISFGWESQPIR | 68 |
| 820–833 | 1591.7154 | 1592.5734 | +0.1493 | SYGEFYDDGISALR | 91 |
| 845–865 | 2312.1397 | 2312.9695 | +0.1775 | SQLEHLEGTPTIWNTSTAATR | 154 |
