# Supplementary material for: Characterization of a Botybirnavirus Conferring Hypovirulence in the Phytopathogenic Fungus Botryosphaeria dothidea
Source: Viruses. 2019 Mar 17;11(3):266. doi: 10.3390/v11030266 (PMC6466033; doi:10.3390/v11030266)
Supplement: Supplementary file 1 [file viruses-11-00266-s001.zip › viruses-449497-supplementary/Manuscript supplementary Figure 5.doc]

**Supplementary**

**Figure S5.** Genomic organization schematic diagrams of Botrytis porri botybirnavirus 1 (BpRV1), Bipolaris maydis botybirnavirus 1 strain BdEW220 (BmBRV1-BdEW220), Sclerotinia sclerotiorum botybirnavirus 1 (SsBRV1), and Alternaria botybirnavirus 1 (ABRV1).
